# Interpretable Predicting Creep Rupture Life of Superalloys: Enhanced by Domain‐Specific Knowledge

**DOI:** 10.1002/advs.202307982

**Published:** 2024-01-02

**Authors:** Jiawei Yin, Ziyuan Rao, Dayong Wu, Haopeng Lv, Haikun Ma, Teng Long, Jie Kang, Qian Wang, Yandong Wang, Ru Su

**Affiliations:** ^1^ School of Materials Science and Engineering Hebei University of Science and Technology Shijiazhuang Hebei 050018 China; ^2^ Max‐Planck‐Institut für Eisenforschung 40237 Düsseldorf Germany; ^3^ School of Materials Science & Engineering Shandong University Jingshi Road 17923 Jinan 250061 China; ^4^ State Key Laboratory for Advanced Metals and Materials University of Science and Technology Beijing Beijing 100083 China

**Keywords:** creep rupture life prediction, heat treatment, machine learning, superalloy

## Abstract

Evaluating and understanding the effect of manufacturing processes on the creep performance in superalloys poses a significant challenge due to the intricate composition involved. This study presents a machine‐learning strategy capable of evaluating the effect of the heat treatment process on the creep performance of superalloys and predicting creep rupture life with high accuracy. This approach integrates classification and regression models with domain‐specific knowledge. The physical constraints lead to significantly enhanced prediction accuracy of the classification and regression models. Moreover, the heat treatment process is evaluated as the most important descriptor by integrating machine learning with superalloy creep theory. The heat treatment design of Waspaloy alloy is used as the experimental validation. The improved heat treatment leads to a significant enhancement in creep performance (5.5 times higher than the previous study). The research provides novel insights for enhancing the precision of predicting creep rupture life in superalloys, with the potential to broaden its applicability to the study of the effects of heat treatment processes on other properties. Furthermore, it offers auxiliary support for the utilization of machine learning in the design of heat treatment processes of superalloys.

## Graphical abstract

The workflow in the above figure is used to show the framework and how to use it to achieve HT optimization with enhanced creep rupture life. Figure (a) presents the model process, i.e., label HT, evaluate HT, and creep rupture prediction. Figure (b) shows the experimental validation for HT optimization based on the three processes.

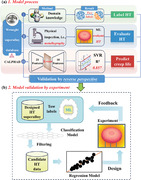

## 1 Introduction

Due to exceptional properties at high temperatures, Ni‐based wrought superalloys have become essential materials in aero‐engine manufacturing.^[^ ^1^ ^]^ During their service, creep rupture is one of the main failure forms.^[^ ^2^ ^]^ Consequently, conducting thorough evaluations of creep rupture life becomes crucial for achieving scientifically robust designs and ensuring reliable and safe service of superalloys. However, determining creep life data through experimental methods is time‐consuming and costly.^[^ ^3^ ^]^ Several theoretical approaches, e.g., time‐temperature parameters methods^[^ ^4^ ^]^ and creep constitutive models,^[^ ^5^ ^]^ have been proposed to accelerate the creep rupture life prediction and show promise in this field. Nevertheless, evaluating creep rupture life necessitates the consideration of complex factors, such as alloy composition, molding processes, heat treatment (HT) conditions, and environmental influences. As a result, the conventional approaches suffer from limitations including inadequate predictive accuracy, insufficient microstructural understanding, and incomplete microstructure evolution considerations.^[^ ^4^ ^]^

The rapid progress in computational science has promoted machine learning (ML) as the preferred approach for revealing the complex relationships between material characteristics and relevant properties.^[^ ^5^ ^]^ In recent years, ML has made remarkable achievements in predicting creep rupture life for superalloys. For example, Venkatesh et al.^[^ ^6^ ^]^ successfully achieved precise predictions exceeding 90% for the creep life of single crystal superalloys by incorporating pertinent material features into neural network models. Shin et al.^[^ ^7^ ^]^ were pioneers in integrating a high‐throughput computational thermodynamic approach with ML for the prediction of creep performance in superalloys. They utilized Pearson's correlation coefficient and maximal information coefficient analysis to identify stress and creep test temperature as the primary influencing features for the creep performance. Furthermore, Han et al.^[^ ^8^ ^]^ innovatively deduced creep fracture life by adjusting the predicted test stress, introducing novel insights for creep life prediction. However, the majority of previous research only used alloy compositions and processing conditions as the ML descriptors, which limits the generalization of ML models due to the lack of physical constraints in the “black box” of the models.^[^ ^9^ ^]^ Zhu et al.^[^ ^10^ ^]^ attempted to utilize atomistic‐level physical features, e.g., atomic radius/volume and electronegativity, to predict the creep rupture life of superalloys. Nevertheless, this approach lacks research on creep damage mechanisms, resulting in insufficient model interpretability.

Moreover, most of the previous researches only depend on ML models consisting of statistical learning methods, i.e., fitting the non‐linear relationship between the composition and the creep performance. These models lack the incorporation of enough physical constraints from the domain‐specific knowledge. Even in practical studies, there is a lack of adequate analysis of the HT process.^[^ ^11^ ^]^ From the ML perspective, the use of high‐quality, high‐dimensional descriptors, coupled with robust domain‐specific knowledge as input, can significantly mitigate the issue of overfitting and boost the predictive accuracy of the model commonly encountered in contemporary ML methods,^[^ ^12^ ^]^ including neural networks with an excessive number of neurons. Furthermore, the successful evaluation of HT's influence on creep rupture life can assist ML models in distinguishing the relationship between various HT processes and creep performance, thereby enhancing prediction accuracy. From the materials science perspective, the creep performance can be significantly improved by adjusting HT parameters such as heating temperature^[^ ^13^ ^]^ and cooling method,^[^ ^14^ ^]^ accompanied by different creep mechanisms. And these process parameters can be optimized effectively by powerful interpretable ML models in conjunction with optimization algorithms such as Bayesian Optimization. However, to date, there is a notable absence of research in the field of utilizing ML to evaluate the influence of HT on the creep rupture life of superalloys. This is due to the fact that this task is often considered one of the costliest endeavors, requiring the acquisition of reliable labeled data followed by iterative improvements through ML. As a result, this endeavor has not yet garnered attention in the relevant domain.

**Figure 1:**
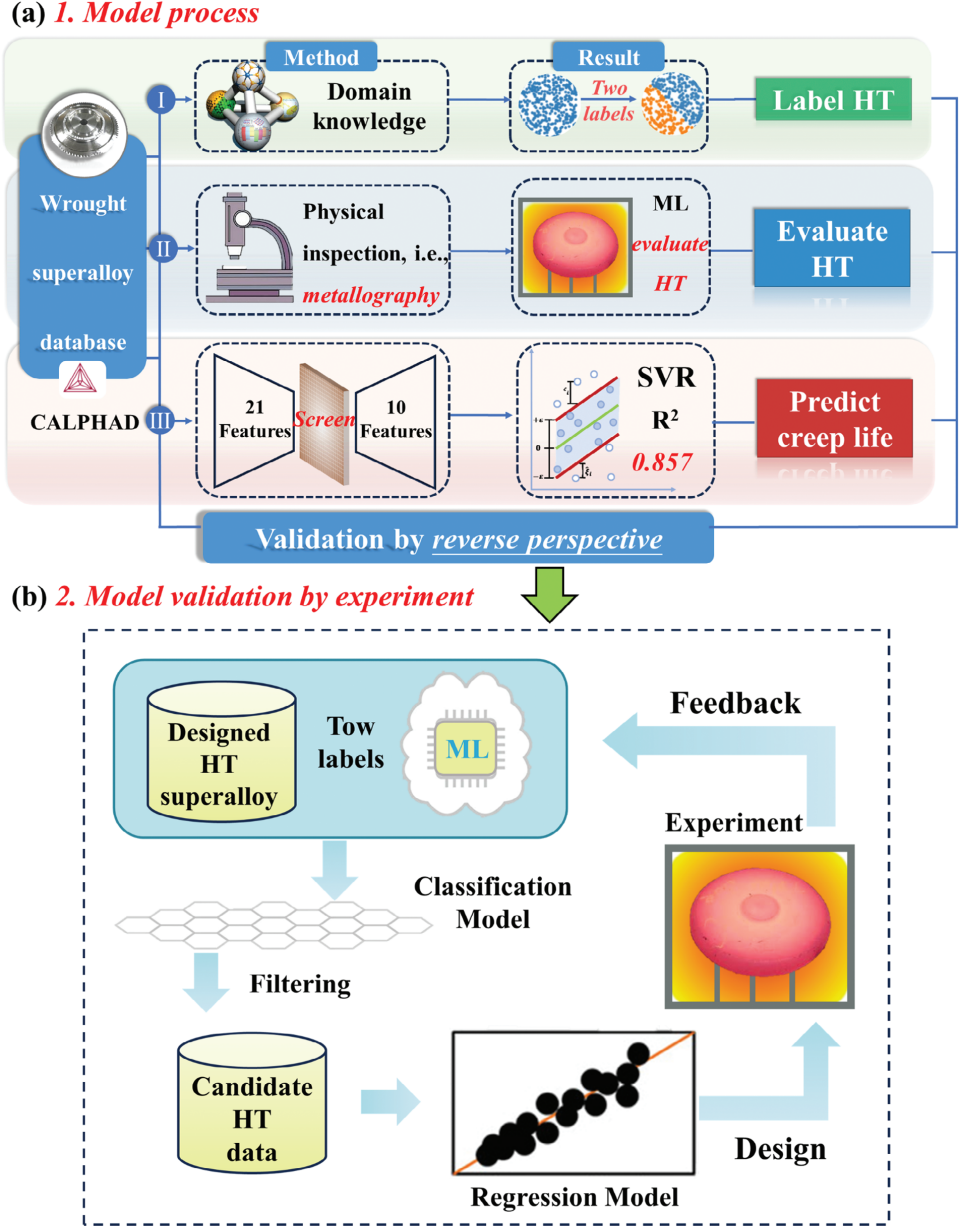
Approach overview. a) We presented a workflow framework for ML‐based prediction of creep rupture life and evaluation of HT processes. The framework consists of three primary steps: (I) Evaluating HT via domain‐specific knowledge; (II) Evaluating HT via a physics‐informed classification model and (III) Predicting creep rupture life by regression models enhanced by screening key features. SVR represents support vector regression. b) In the experimental validation phase, initially, we employ a classification model embedding physical features for the preliminary evaluation and screening of the designed HT. To determine which HT process can achieve the optimal creep life, we utilize a regression model (after screening features) to predict the creep life. The ultimate goal is to achieve process optimization with extremely low experimental costs.

To address this research gap, our study investigates the relationship between HT and the creep performance of superalloys with interpretable classification and regression models. To achieve this goal, in addition to the basic features, we utilized the CALPHAD (CALculation of PHAse Diagrams) method to introduce domain‐specific knowledge, i.e., the dissolution temperature and the size of γ′/γ′′ phases and the volume fraction of δ phase. Simultaneously, four types of physical features, reflecting creep performance and accounting for process‐structure‐performance relationships, were collected and used to evaluate HT. The regression model's coefficient of determination improved from 0.612 to 0.857 and significant improvements were also obtained in the evaluation indexes of the classification model. Using this ML approach to optimize the HT, the newly acquired HT results in significant improvement compared to previous studies (**Figure** **1**).

## 2 Results

We use the workflow in Figure 1 to show our framework and how to use it to achieve HT optimization with enhanced creep rupture life. Figure 1a presents the model process, i.e., label HT, evaluate HT, and creep rupture prediction. Figure 1b shows the experimental validation for HT optimization based on the three processes.

The details are shown below:

1) Label HT: we first partitioned the original dataset into two subsets, i.e., dataset 1 and dataset 2, with domain‐specific knowledge. Dataset 1 contains HT processes that positively affect creep life (Labeling it as 1), whereas Dataset 2 (Labeling it as 0) does not. We further evaluate the prediction performance of the different ML models on these two datasets. Dataset 1 exhibits superior predictive performance, a quality attributed to the inclusion of crucial features and the precise application of domain knowledge. In contrast, dataset 2 demonstrates relatively poorer performance in prediction. This phenomenon may be attributed to lower correlations between the features and creep rupture life or other unaccounted factors.
2) Evaluate HT: obtaining labeled high‐quality data is a costly step in ML. Utilizing ML models to label and incrementally augment unlabeled HT data is crucial for superalloy development. Establishing a suitable classification model is paramount, considering the limited availability of datasets, potential lower accuracy, and constrained extrapolation capabilities in direct ML applications. For effective HT evaluation, we employed distinct classification models for each selected alloy to achieve optimal performance. Moreover, acknowledging the typical “black‐box” nature of ML models, we introduced key physical features based on the process‐structure‐performance relationship. This not only aids in evaluating HT processes but also enhances model interpretability, striking a balance between physics‐based constraints and the intricate correlations behind ML.
3) Creep rupture prediction: After the preliminary evaluation of HT, candidate data is obtained. Given the high cost of creep experiments, we further utilize regression models and unsupervised learning methods to screen these candidate HT processes. To accurately predict creep rupture life, we employ key feature selection methods in the predictive model based on the Pearson correlation coefficient and exhaustive search. Based on the finding in process 1, we contend that utilizing dataset 1 for predicting the creep rupture life holds greater practical and research significance. Our method allows us to pinpoint the key alloy components and process parameters for prediction from a multitude of features. Our key feature screening methodology significantly improves the accuracy of the model for predicting creep rupture life.
4) Experimental validation: we use the HT design of Waspaloy alloy as an example. Initially, we employ a classification model embedding physical features for the preliminary evaluation and screening of the designed HT. To determine which HT process can achieve the optimal creep life, we utilize a regression model (after screening features) to predict the creep life. The ultimate goal is to achieve process optimization with extremely low experimental costs. The improved HT led to a significant enhancement in creep performance (5.5 times higher than the previous study).

### 2.1 Labeling HT Processes on Creep Rupture Life Prediction and Evaluation of HT by Transfer Learning

**Figure 2:**
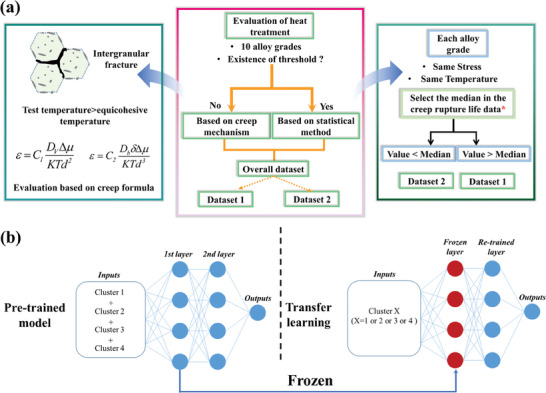
a) Schematic of the procedure of the evaluation of the HT process via specific knowledge. By using two methods, the overall dataset was divided into two datasets, dataset 1 and dataset 2. b) Schematic diagram of transfer learning for evaluating HT of superalloy using total data transfer.

To conduct an initial evaluation of the HT process and facilitate subsequent classification and regression models, we use two methods (i.e., statistical method and mechanism method) to manually classify the dataset to be dataset 1 and dataset 2 as shown in **Figure** **2**a. The details of the evaluation are constructed as follows: First, we evaluate whether there exist thresholds for the same alloy type under approximate creep conditions, if yes, we use the statistical method, if not, we use the mechanism method. Second, if we use statistical methods, the threshold value for creep rupture life is selected as the median. Third, if we use the mechanism method, the HT is evaluated by analyzing whether the microstructure of the alloy is suitable for the creep test conditions. More details are elaborated in Section S2 (Supporting Information). In summary, we used the above methods to evaluate the effect of the HT process on creep rupture life and divided the dataset into two datasets.

As shown in **Table** **1**, we conducted prediction by using five ML regression models: Random Forest (RF), Gradient Boosting Regressor (GBR), Multi‐Layer Perceptron Regression (MLPR), Linear Regression (LR), and SVR. The SVR model achieved the most accurate predictions and was not overfitting. Additionally, as indicated in **Table** **2**, when predicting each dataset separately, notable improvements were observed in the predictions for Dataset 1 compared to Dataset 2. From a practical standpoint, utilizing dataset 1 for predicting the creep life of superalloys not only yields more reliable results but also highlights the positive effects of the included HT on creep performance. Moreover, leveraging the foundation of the following classification model allows for further refinement of suitable HT processes. Therefore, predicting the creep life of superalloys using dataset 1 makes more sense. The superior predictive performance of dataset 1 also underscores the indispensable nature of domain‐specific expertise in ML.

**Table 1:** Overall prediction performance of the five ML models for the two datasets (tenfold cross‐validation).

Candidate models	R^2^‐Ave	R^2^‐Dev	RMSE‐Ave	RMSE‐Dev
**SVR**	0.676	0.151	0.535	0.507
**RF**	0.582	0.122	0.747	1.012
**GBR**	0.489	0.236	0.791	0.722
**MLPR**	0.488	0.153	0.787	1.122
**LR**	0.438	0.172	0.786	0.915

*Ave = Average, Dev = Deviation.

**Table 2:** The prediction of the SVR for dataset 1 and dataset 2.

Score Dataset	R^2^	Error
**1‐Ave**	0.612	6.45%
**1‐Dev**	0.151	2.35%
**2‐Ave**	0.405	14.39%
**2‐Dev**	0.272	12.75%

Given that ML can automatically evaluate HT processes undoubtedly enhances efficiency compared to manual annotation. However, there is significant variation in the scale of HT data for each alloy, with some alloys having extremely limited available datasets. Therefore, applying ML algorithms directly to such small datasets for HT is not feasible. To overcome this challenge, we carefully considered transfer learning (TL) as a solution,^[^ ^15^ ^]^ believing in its effectiveness in retaining useful features from the pre‐trained model to other individual tasks. We meticulously selected 208 data points from the entire dataset, representing four distinct alloy types (also referred to as clusters, totaling 4 clusters). The specific selection criteria are detailed in Section S3 (Supporting Information). Through this framework shown in Figure 2b, we first pre‐trained a classification model using all data from cluster 1 to cluster 4. After that, in the TL, we freeze the first layer of the model, and only re‐train the second layer, aiming to retain useful features from the pre‐trained model for better adaptation to different clusters. We commence the model training process by pre‐training on an extensive dataset, encompassing clusters 1, 2, 3, and 4. This initial phase is crucial to glean a comprehensive understanding of diverse patterns and features present in the data. Subsequently, armed with the insights gained from the pre‐training phase, we embark on the fine‐tuning task on clusters 1 to 4, respectively. The final predictions are shown in **Table** **3**, for clusters 1 and 2, both the training and validation accuracies are relatively high, while the overall performance of clusters 3 and 4 is comparatively poor. Overall, the performance of Cluster 1 and Cluster 2 in transfer learning is better than that of Cluster 3 and Cluster 4. Given the unsatisfactory predictive performance of the TL method, we subsequently adopted an approach of establishing separate classification models for each data cluster to explore the evaluation of HT by ML in our next section of work.

**Table 3:** Prediction results for each cluster were obtained by using all data for pre‐training through transfer learning. (The ratio of the training set to the testing set is 4:1).

Data cluster	models	Evaluation scores for the model		
		Validation accuracy (tenfold)	Accuracy (testing set)	Auc values (testing set)
**All alloys**	***Pre‐trained***	71.21%	76.19%	81.40%
**Cluster 1**	Transfer learning	71.43%	77.60%	95.80%
**Cluster 2**	Transfer learning	72.22%	68.18%	95.83%
**Cluster 3**	Transfer learning	40.00%	66.70%	88.90%
**Cluster 4**	Transfer learning	40.00%	63.30%	77.70%

### 2.2 Evaluation of HT Processes by Supervised ML Models

Considering the suboptimal predictive performance of the TL method, we directly employ a supervised learning approach for prediction in this section. Obtained classification results were analyzed and presented in **Table** **4** (for detailed prediction results, refer to Section S3, Supporting Information). According to the no free lunch theorem, five different ML models were employed for each model. By comprehensively evaluating the accuracy and Auc values, we selected the best model from SVC (Support vector classifier), RFC (Random‐forest classifier), DTC (Decision tree classifier), MLPC (Multi‐layer perceptron classifier), and BC (Bagging Classifier) models. As shown in **Table** **5**, DTC, MLPC, and BC show the best results and were therefore chosen as the optimal models. The results indicated that cluster 2 demonstrated the best predictive performance, followed by cluster 3. However, clusters 1 and 4 predict ion performance as subpar, with an accuracy of only 70%, and an Auc value even lower than 0.8. This implies that the model prediction capabilities do not have enough practical value. The initial results highlighted imbalances in predictive outcomes among the various clusters and shortcomings in predictive accuracy.

**Table 4:** Prediction results for each data cluster without added physical features.

Data cluster	Optimal model	Evaluation scores for the model		
		Accuracy (tenfold)	Accuracy (testing set)	Auc value (testing set)
**All alloys**	/	79.70%	80.95%	0.82
**Cluster 1**	DTC	63.33%	75.00%	0.73
**Cluster 2**	MLPC	86.36%	86.36%	0.87
**Cluster 3**	BC	86.00%	83.33%	0.83
**Cluster 4**	BC	70.83%	66.67%	0.75

*The data clusters from 1 to 4 represent Waspaloy, IN718, U720Li, and AD730 alloy.

**Table 5:** Inputs, outputs labels, and indicators for model evaluation used in this ML approach.

	Model	Inputs and outputs	Indicators for model evaluation
Inputs	Regression model	Composition	R^2^ RMSE MAPE
		HT parameters	
		Testing condition	
Outputs		Creep rupture life	
Inputs	Classification model	Composition	Accuracy Precision Recall F1‐score Auc value
		HT parameters	
		Testing condition	
		Physical features	
Outputs		HT evaluation	

R^2^, RMSE, and MAPE represent the coefficient of determination, Root Mean Square Error, and Mean Absolute Percentage Error, respectively.

**Figure 3:**
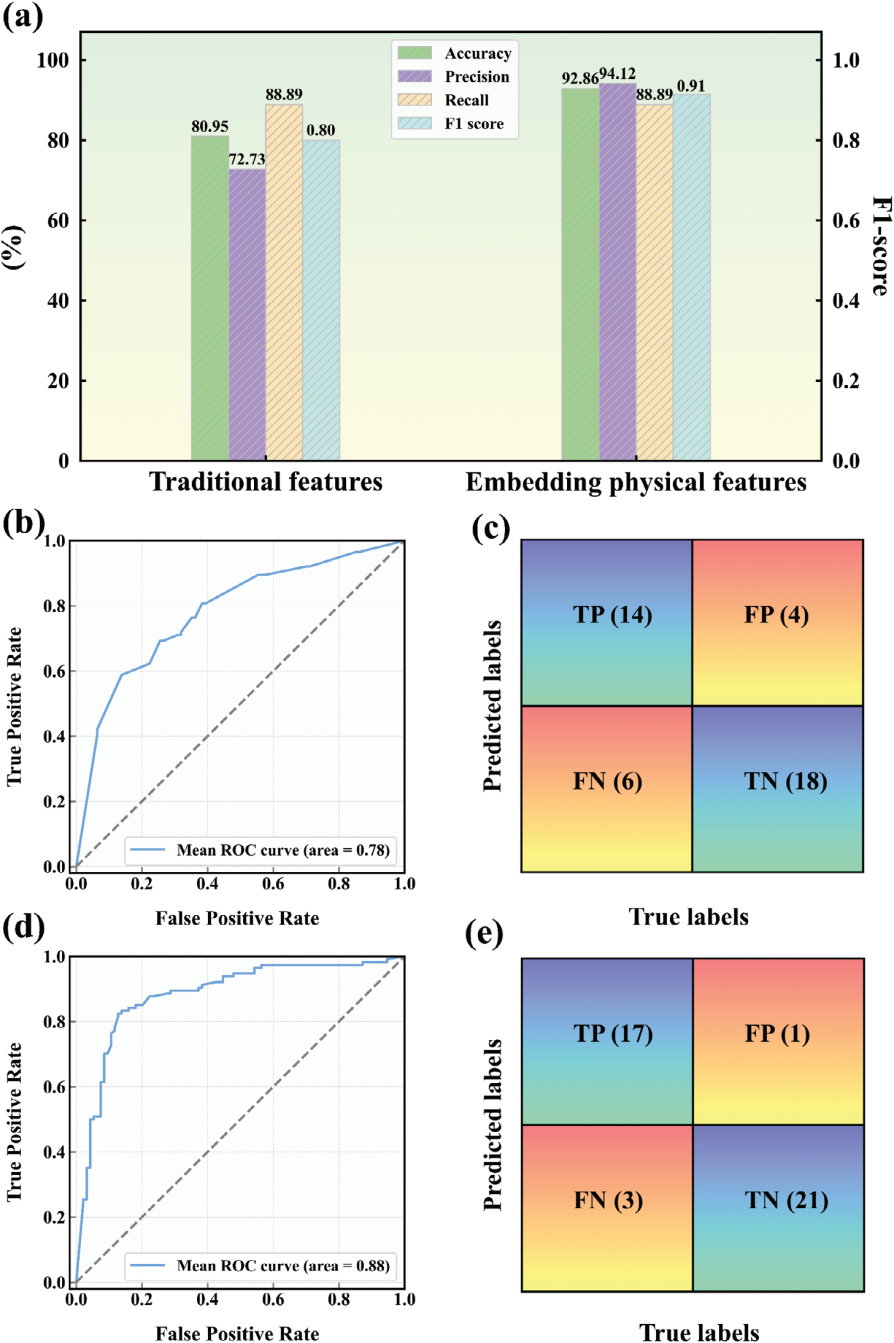
a) Comparison of prediction results of the testing set between models without and with the addition of new features; b) Roc plots for training data classification prediction without embedding physical features (tenfold cross‐validation); c) Confusion matrix for models without physical features; d) Roc plots for training data classification prediction with physical features (tenfold cross‐validation); e) Confusion matrix for models with physical features.

After additional physical features were incorporated (The physical features added for each data cluster are presented in Table S3, Supporting Information), the improved predictions resulting from the inclusion of these new features are presented in **Table** **6** Remarkably, the performance of the model significantly improved after the integration of these additional features. As shown in Table 6, after incorporating the physical features, DTC, MLPC, and RFC demonstrated the best results and were therefore chosen as the optimal models. Among them, cluster 3 showed the most notable improvement, which had a prediction accuracy of 100% on its testing set. These results underscore the enhanced reliability and practical value of the classification model following the incorporation of these physical features. Furthermore, a comprehensive comparison was conducted between the predictions of all testing sets, as illustrated in **Figure** **3**a–e. The evaluation metrics, including accuracy, precision, recall, and F1‐score, demonstrated substantial improvements over the previous model upon integrating the new features. Moreover, there was a notable enhancement in the percentages of TP and TN, and the area of the Roc curve is significantly improved with the addition of physical features. These findings reinforced the influential role of the new features in the classification model, affirming the reliability of our classification modeling approach and presenting novel insights for the application of ML techniques in the field of superalloys. The predictive results demonstrated the crucial role of the physical features obtained from experiments in ML models. However, acquiring a substantial amount of usable physical feature data through experiments is both time‐consuming and expensive. Hence, utilizing a small amount of experimental data to train ML models for predicting the required physical features emerges as a potential solution. Taking grain size after HT as an example, currently, it can only be acquired through physical inspection, i.e., metallography. Consequently, we employed an ML model to predict post‐HT grain sizes (for detailed prediction results, refer to Section S5, Supporting Information).

**Table 6:** Prediction results for each data cluster with added physical features.

Data Cluster	Optimal model	Evaluation scores for the model		
		Accuracy (tenfold)	Accuracy (testing set)	Auc value (testing set)
**All alloys**	/	85.88%	92.85%	0.91
**Cluster 1**	MLPC	82.50%	87.50%	0.83
**Cluster 2**	DTC	87.51%	95.45%	0.93
**Cluster 3**	MLPC	83.33%	100.00%	1.00
**Cluster 4**	RFC	80.00%	83.33%	0.88

### 2.3 Improve Creep Rupture Life Prediction via Screening Key Features

**Figure 4:**
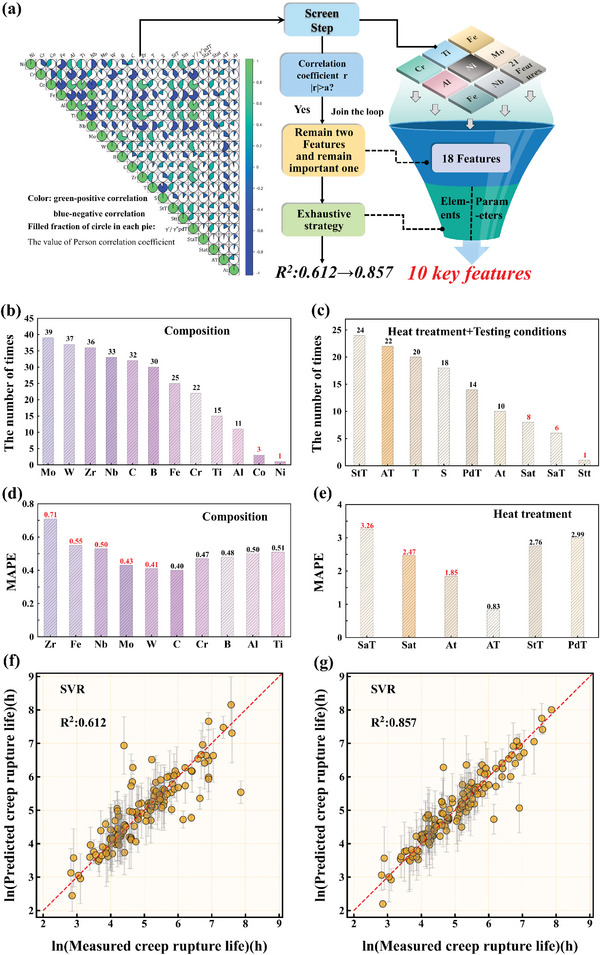
a) Steps for screening features based on the SVR model. b,c) Feature screening results of the second step (StT represents “AT” and “r” stands for the solution treatment temperature, aging temperature, and correlation coefficient. PdT is an abbreviation for γ″/γ″′ phases dissolution temperature); d,e) Feature screening results of the third step. f,g) Predictive performance of the SVR on dataset 1 (before and after the screening of key features) (tenfold cross‐validation).

To improve the prediction accuracy of the model and identify the key features that influence creep rupture life. Consider that the Pearson correlation coefficient can effectively quantify the linear relationship between continuous variables and systematically explore the entire parameter space. As shown in **Figure** **4**a, in the first step, we traversed the Pearson correlation coefficients (r) per feature, utilizing the median (a) as the threshold for assessing the inter‐feature correlations. Only when the correlation coefficient exceeded the threshold (|r| > a) did we include that feature in the construction of the regression model and evaluate its impact on the MAPE. In the second step, the retention frequency of each feature was counted following the completion of the nested for loops. These features were categorized into two distinct subsets to differentiate their influence on alloy composition and HT process parameters. Furthermore, during the process, features that appeared fewer than five times were excluded. This filtering measure was implemented because they typically exert a significant impact on creep performance, e.g., SaT (stable aging temperature) and Sat (stable aging time), among others, despite some features having a low frequency in feature retention. Due to the limitations of a single screening method, some crucial features might be mistakenly omitted. In the third step, we retained experimental temperature and stress variables, while the remaining 16 features underwent exhaustive screening for composition and process conditions using the SVR model. Model construction commenced with the two feature sets retained from the second step, progressively removing one feature in each iteration. This process continued until the termination condition was met. After each iteration, we retained only those features that significantly reduced the MAPE value. Ultimately, we identified ten key features that exerted a significant influence on creep life.

Figure 4b–e shows the feature screening results based on the screening strategy. In the second step, Ni and Co are excluded along with the Stt (solid solution treatment time). In the third step, the alloy composition was refined by removing Fe, Nb, Mo, W, and Zr. Furthermore, SaT, Sat, and At (aging time) from the HT parameters were also eliminated. Based on the discussion above, ten key features were identified. The predictive performance of the model was then evaluated using these ten key features to predict creep rupture life, yielding notably enhanced results. As shown in Figure 4f,g, the coefficient of determination (R^2^) value of the model was improved from 0.612 to 0.857. This finding underscores the efficacy of our key feature screening methodology in significantly improving the accuracy of the model for predicting creep rupture life in superalloys.

### 2.4 Full‐Flow Validation of ML Approach with Experiments

To optimize the HT process of superalloys, a series of HT processes have been designed based on Waspaloy alloy. By using the ML approach, various schemes were validated, and the model‐predicted results guided the optimal selection of the process. In this design, as shown in **Table** **7**, we devised a new HT for the Waspaloy. (Detailed information on the experiment can be found in Section S5, Supporting Information)

**Table 7:** Newly designed HT for Waspaloy.

Number	StT [°C]	Stt [h]	SaT [°C]	Sat [h]	AT [°C]	At [h]
1	1010	4	0	0	760	16
2	1020	4	0	0	760	16
3	1030	4	0	0	760	16
4	1040	4	0	0	760	16
5	1060	4	0	0	760	16
6	1080	4	0	0	760	16

**Figure 5:**
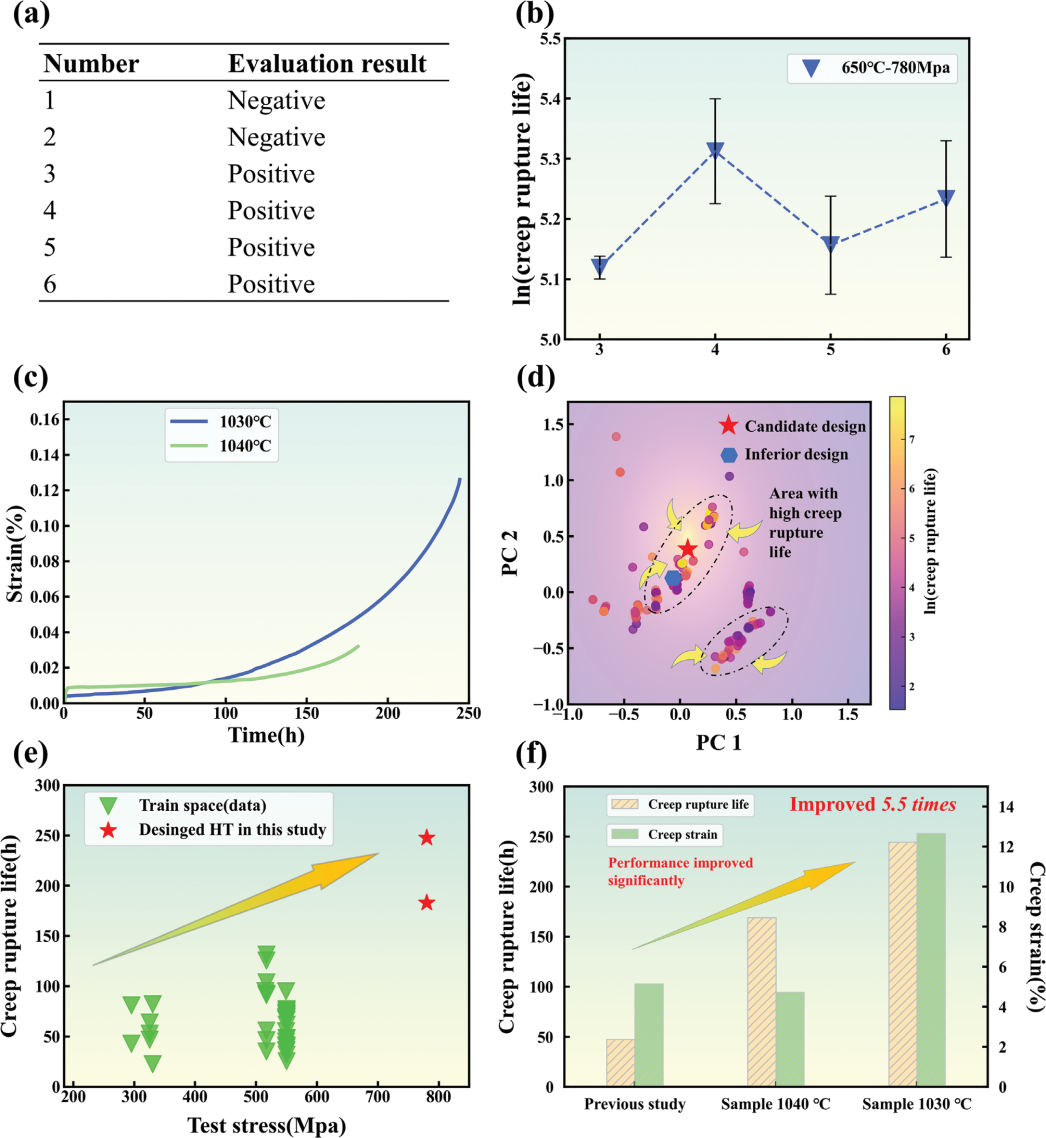
a) Results of a preliminary evaluation of potential designs (Optimization of HT based on Waspaloy alloy with specific process parameters detailed in Table 7). b) Further prediction of potential targets through a regression model. c) Variation curves of the creep strain to the creep time d) The visualization of the HT parameters in a 2D space. The color bar represents the value of ln (Creep rupture life). And the red pentagram shows the position of the candidate process when mapped to 2D space. e) Illustration of the proposed new design alloy candidate for the application of a high‐stress service environment. f) Comparison of the performance of the newly designed process with the previous study.

In **Figure** **5**, we illustrate the validation process and final results of the heat treatment optimization using the machine learning approach. In Figure 5a, we employed a classification model with embedded physical features to conduct a preliminary assessment of candidates for the Waspaloy alloy. The results indicate that heat treatment processes beyond sample 3, with a solution treatment temperature exceeding 1020 °C, positively impact creep performance. Subsequently, in Figure 5b, after feature selection for creep life prediction using a regression model, we observed a turning point in the performance curve starting from sample 4. Due to inherent predictive errors in the model, we have not conclusively determined which heat treatment process is superior. Following this, we performed dimensionality reduction and visualization on all candidate and training data (refer to Figure 5c). The yellow region represents heat treatment processes with high creep rupture life, with only samples 3 and 4 (red pentagrams) falling within the central area. The exclusion of samples 5 and 6 (blue pentagons) may be related to the fact that the structure obtained by the heat treatment process is not the optimal solution under the tested conditions. The experimental results for the final selected two samples are shown in Figure 5d. As depicted in Figure 5e,f, the results demonstrate a significant improvement in creep performance with the enhanced heat treatment process, with sample 3 being particularly noteworthy. This comprehensive validation ensures the feasibility of our final selection in practical applications, leading to substantial performance improvements in engineering applications.

## 3 Discussion

### 3.1 The Role of New Features for Classification Models

**Figure 6:**
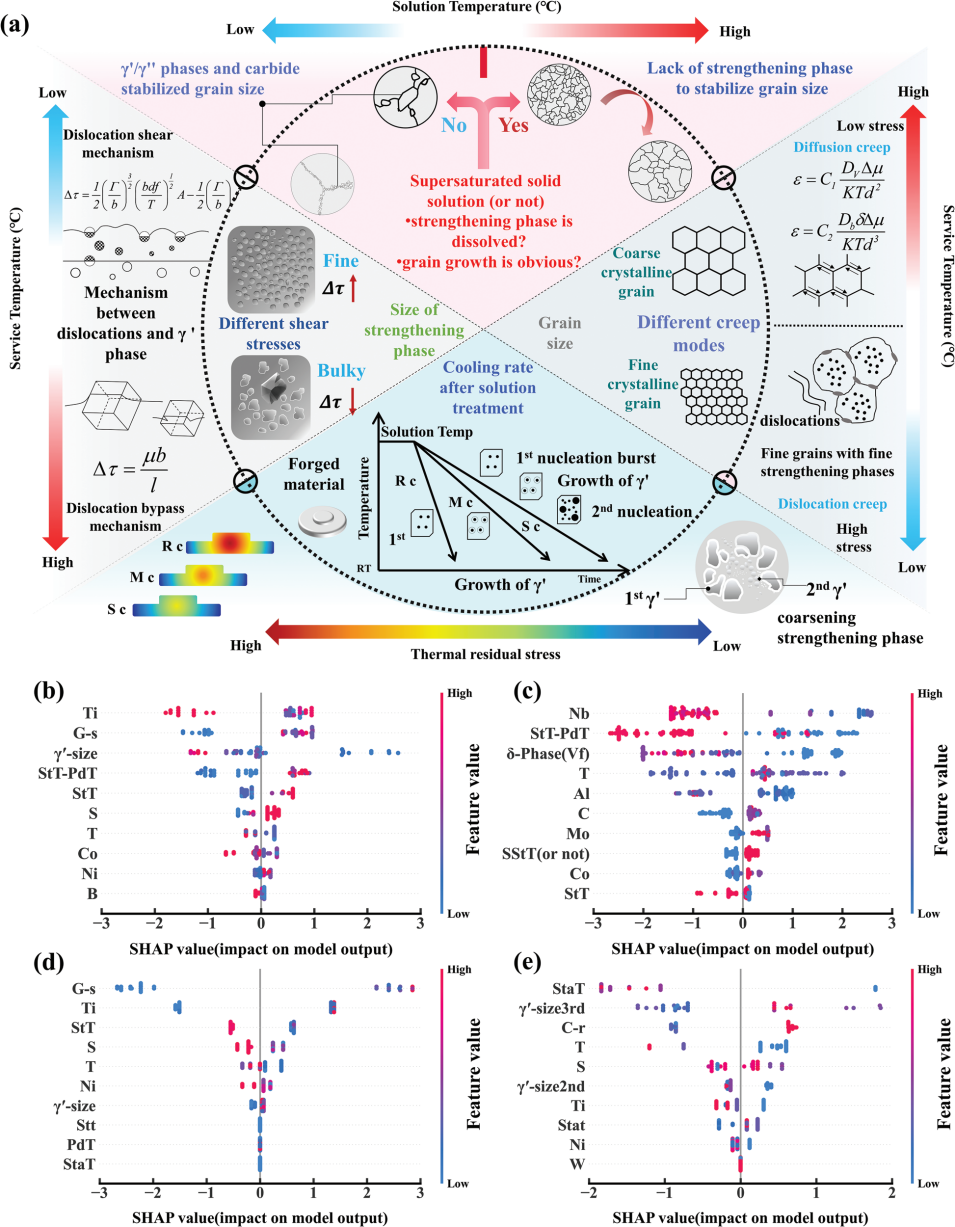
a) New physical features added to data clusters. Rc, Mc, and Sc, respectively, represent the cooling rates of different cooling methods (corresponding to rapid, medium, and slow cooling). For example, methods such as water cooling, air cooling, and furnace cooling. b) Cluster 1: SHAP value summary plot; c) Cluster 2: SHAP value summary plot; d) Cluster 3: SHAP value summary plot; e) Cluster 4: SHAP value summary plot. G‐s presents grain size.

Conventional ML adopts a “black‐box” modeling approach, which makes it difficult to reveal the intrinsic mechanisms of performance and structure. Introducing physical features into the ML model to achieve outstanding performance has been validated in frontier research.^[^ ^17^ ^]^ Therefore, as shown in **Figure** **6**a, we based the ML method on the relationships between process–structure–performance, when considering the addition of new features, the main aspects to take into account are as follows:

First, as depicted in the pink region shown in Figure 6a, the StT is a crucial step in regulating the grain size as well as the size and distribution of γ′/γ′′ phases. When the StT exceeds the dissolution temperature of the γ′/γ′′ phases, the grains lose the pinning effect of the strengthening phase and exhibit significant growth. Therefore, when the solid StT exceeds the dissolution temperature of the γ′/γ′′ phases, (Ordinal Encoding is employed) it is labeled as 1, while it is labeled as 0 when it does not exceed the dissolution temperature. Additionally, the temperature difference between the solid solution temperature and the strengthening phase temperature (StT‐PdT) was introduced to enhance differentiation between data points and improve prediction accuracy.

Second, grain size plays a pivotal role in influencing polycrystalline alloy creep properties. As indicated in the gray region on the right side of Figure 6a, the creep performance of superalloys exhibits a high sensitivity to grain size, and the HT process offers an effective means to precisely control grain size for enhancing alloy performance. Typically, fine‐grain strengthening is considered a conventional method for strengthening alloys during low‐temperature service. However, it is important to note that fine grain structure does not universally enhance material strength under all conditions.^[^ ^18^ ^]^ Researches indicate that with the increase in material testing temperature, the efficacy of grain refinement in improving material strength gradually diminishes.^[^ ^19^ ^]^ This phenomenon arises because, when the experimental temperature is equal to or higher than the equicohesive temperature, the strength within the grains exceeds that at the grain boundaries, causing grain refinement to undermine the alloy performance. Moreover, during creep experiments, alloy grain boundaries experience sliding, leading to fracture propagation along grain boundaries.^[^ ^20^ ^]^ Therefore, considering grain size as a feature input is essential for evaluating the impact of HT processes on creep properties.

Third, as indicated in the gray region on the left side of Figure 6a, the creep resistance of the superalloys depends mainly on the hindrance of the motion of γ′ phase relative to the dislocations. Creep deformation is mainly driven by the interaction between dislocations, which move along different slip system directions intersecting with the γ′ phase, including the dislocation shearing and Orowan by‐passing mechanisms.^[^ ^1^ ^]^ The strengthening effect of the γ' phase relative to the alloy is generally estimated using the critical resolved shear stress (CRSS) ∆τ. When the γ′/γ′′ phases particles are under critical size, they can be sheared or deformed by dislocations, including weakly coupled dislocations for shear and strongly coupled dislocations for shear. For small particles, the CRSS is determined by the stress necessary to move weakly coupled dislocation pairs, according to Equation (1)^[^ ^21^ ^]^:

(1) $$\Delta \tau =\frac{1}{2}{\left(\frac{\Gamma }{b}\right)}^{\frac{3}{2}}{\left(\frac{bdf}{T}\right)}^{\frac{1}{2}}A-\frac{1}{2}\left(\frac{\Gamma }{b}\right)f$$

where Γ is the anti‐phase boundary energy of the γ′ in the {111} plane, *b* is the burgers vector of the edge dislocation in the γ matrix, *d* the γ′/γ′′ phases particle diameter, *f* the volume fraction of the γ′/γ′′ phases precipitates, *T* the line tension of the dislocation, and *A* a numerical factor depending on the morphology of the particles. For spherical particles *A* = 0.72.

For larger γ′/γ′′ phases particles, where dislocations cut in strongly coupled pairs, the CRSS is given by Equation (2)^[^ ^22^ ^]^:

(2) $$\Delta \tau =\left(\frac{1}{2}\right)1.72\frac{Tf^{1/2}}{\mathrm{bd}}{\left(1.28\frac{d\Gamma }{wT}\right)}^{\frac{1}{2}}$$

where *w* is a constant which accounts for the elastic repulsion between the strongly paired dislocations, and which is of the order of unity.

Conversely, if the precipitate size is larger than the critical size, the strong dislocation pair can cut through or bypass precipitates by Equation (3)^[^ ^23^ ^]^:

(3) $$\Delta \tau =\frac{\mu b}{l}$$

where *µ* is the shear modulus, *l* is the particle phase spacing, that is, when the particle phase size increases, the spacing *l* also increases.

When the γ′/γ′′ phase size does not exceed the critical size, an increase in the size of strengthening phase particles and volume fraction effectively enhances their ability to resist dislocation shear. However, excessively large particle sizes weaken their hindering effect on dislocations. Therefore, well‐dispersed fine γ′/γ′′ phases achieve significant strengthening effects. It provides sufficient strengthening effects when the size is below the critical size and, during the creep deformation, continues to moderately slow down the coarsening rate of the strengthening phase, thereby enhancing creep resistance. Therefore, introducing the size of the γ′/γ′′ phase particles as a physical feature would help the ML model effectively evaluate the effect of the HT process on creep performance.

Fourth, as shown in the blue indigo part of Figure 6a, the cooling rate influences the precipitation process, morphology, and size of the γ′/γ′′ phases.^[^ ^24^ ^]^ A faster cooling rate promotes the precipitation of spherical and finely distributed strengthening phases, resulting in enhanced alloy strengthening. However, an excessively rapid cooling rate can lead to thermal residual stress concentration and subsequent deforming cracking.^[^ ^25^ ^]^ Conversely, a slower cooling rate allows for sufficient atom diffusion, leading to the formation of bulk strengthening phases and secondary/tertiary strengthening phases of varying sizes.^[^ ^26^ ^]^ This adversely affects creep properties. Therefore, considering an appropriate cooling rate is crucial for optimizing HT.

The SHAP (SHapley Additive Explanations) method was used to assess the influence of each key feature on the prediction. As shown in Figure 6b–e, ten key features were selected and ranked based on their importance. It is worth noting that most of the newly added physical features have become critical factors influencing the classification model. For cluster 1, the outstanding creep performance is associated with increasing the StT, medium‐sized grains, and refined γ′ phase. In cluster 2, the key to achieving ideal creep properties lies in reducing the volume fraction of the δ phase through HT. For clusters 3 and 4, the parameters related to aging and solution treatment, determine the creep performance. Relatively lower ATs, faster cooling rates, and appropriately sized grains and γ′ phase particles can effectively improve the creep life.

### 3.2 Analysis for Insufficient Prediction of Conventional SVR Model

**Figure 7:**
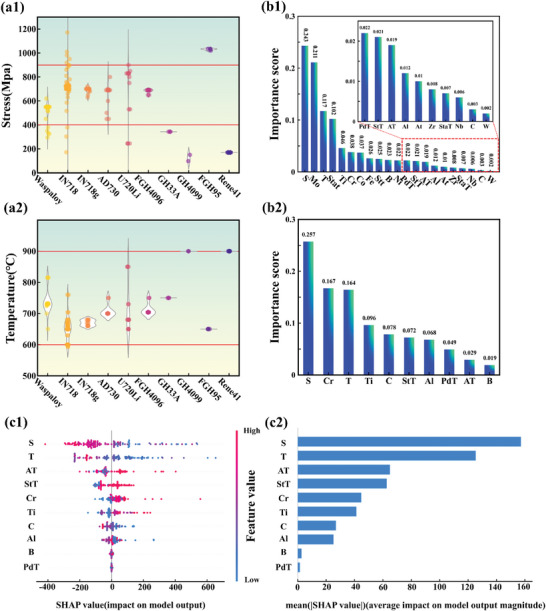
(a1) and (a2) are scatter plots and violin‐type plots for test temperature and stress. b1) The importance ranking of the initial input features (Random Forest); b2) The ranking of feature importance of key features (Random Forest); c1) SHAP value summary plot; c2) mean |SHAP value| bar graph. (All of the above are analyses for dataset 1).

From the perspective of data distribution, as depicted in **Figure** **7**a1,a2, the majority of the data concentrates in the medium to high‐stress and medium to high‐temperature range in creep conditions, exhibiting a significant range of fluctuations. Notably, all outliers are located within this specific data range. Strengthening phase and dislocation interactions in such environments are influenced by shearing, Orowan bypass, and thermally activated climb bypass mechanisms.^[^ ^27^ ^]^ Consequently, different microstructures emerge under different creep conditions characterized by varying combinations of stress and temperature. However, due to the complexity of the creep mechanisms involved, the features present in this dataset fail to adequately represent the inherent variations in creep mechanisms, resulting in significant deviations between predicted and actual measurements.

To further conduct a visual analysis of the input features and based on comprehensiveness and interpretability, we adopted a method that combines random forest feature importance analysis and the SHAP model to demonstrate the impact of input on creep rupture life. Turning to feature engineering, Figure 7b1 shows the ranking of feature importance, among the key alloy compositions, Mo occupies the top position. Traditionally, Mo is primarily employed as a refractory element in superalloys for solid‐solution strengthening.^[^ ^28^ ^]^ However, the collected superalloys in this study rely on precipitation strengthening, which hinges on the addition of Al and Ti with the role of Mo being less apparent. Moreover, some researchers have posited that an excessive concentration of Mo accelerates the precipitation of the TCP phase (topo‐logically closed phase), thereby detrimentally impacting creep rupture life.^[^ ^29^ ^]^

### 3.3 Analysis of Key Feature Screening Method

As shown in Figure 7b2,c2, the random forest importance and the SHAP model were employed. Notably, variations in the importance rankings were observed between the two methods. These discrepancies can be attributed to the different algorithms employed to determine the importance of strengths. Importantly, the goal of importance ranking in this study is not to establish one method as superior, but to explore how different features affect creep rupture life.

Ti, Al, C, and B are primary constituents of the strengthening phases in superalloys. In addition to their role in strengthening phase formation,^[^ ^1^ ^]^ Ti and Al also contribute to solid solution strengthening, thereby increasing the yield strength.^[^ ^30^ ^]^ The effect of elements C and B on the creep resistance of superalloys remains a subject of debate. Generally, the C content in superalloys should be limited to less than ≈0.1 wt.% to avoid detrimental effects on alloy toughness and creep properties caused by the presence of primary carbides (MC).^[^ ^31^ ^]^ B has been found to improve intergranular bonding and increase grain size during creep, thereby significantly enhancing the creep resistance of the alloy.^[^ ^32^ ^]^

However, the influence of the Cr on creep properties exhibits a dual nature. In Ni‐based superalloys, Cr primarily enhances high‐temperature oxidation and corrosion resistance by forming a protective oxide layer on the alloy surface.^[^ ^33^ ^]^ However, at elevated temperatures, atomic‐scale mechanisms cause the aggregation of Cr elements in the dislocation network,^[^ ^34^ ^]^ creating diffusion channels and concentration gradients between the matrix phase and the reinforcing phase. This results in directional coarsening of the reinforcing phase along the elemental concentration gradient, leading to a reduction in the material creep resistance. Conversely, in the study of Co‐based superalloys, Cr plays a role in reducing lattice mismatch, slowing down the coarsening rate of the γ' phase, and enhancing the effect of antiphase boundary energy and the strengthening effect of the γ' phase, thereby improving creep properties.^[^ ^33^, ^35^ ^]^ Moreover, Cr has been reported to have the strongest effects in the stacking faults energy on both the γ and γ' phases which can improve creep property.^[^ ^36^ ^]^ Accurately determining the precise effect of Cr on creep properties remains a challenge. ML results indicated that Cr is considered the most critical element influencing the alloy creep resistance. The advantage of ML lies in its ability to handle complex relationships between targets and features, avoiding misleading results caused by unclear mechanisms. Therefore, in subsequent studies aimed at improving the creep performance of alloys, Cr should be considered one of the most crucial features in modifying alloys. Additionally, Figure 7c1 reveals that the creep properties of the alloy can be effectively enhanced by appropriately increasing the content of Cr and Ti in the alloy composition while controlling the levels of Al, C, and B within reasonable ranges.

The ML model suggests focusing on StT and AT. This is closely linked to the development of superalloy. With an increase in the γ' phase within the alloy,^[^ ^37^ ^]^ adopting a multi‐step aging process often results in the precipitation of coarser strengthening phases, leading to instability in the size and quantity of the γ' phase during service.^[^ ^38^ ^]^ In contrast, single aging facilitates achieving a fine and uniform distribution of strengthening phases within the alloy, increasing the quantity of strengthening phases, and thereby enhancing the alloy's performance. Therefore, in designing HT processes for superalloys at elevated temperatures, it is advisable to simplify aging steps, considering StT and AT as crucial parameters for coordinating alloy structure. By emphasizing these aspects, efforts can be made to improve the design and optimization of superalloy HT processes, thereby enhancing creep resistance and overall performance. Notably, the PdT plays a significant role in determining creep rupture life. Previous investigations have revealed that raising the dissolution temperature of the γ′/γ′′ phases enhances the creep performance of superalloys.^[^ ^39^ ^]^ This improvement allows them to withstand higher Orowan stresses at elevated temperatures and increases the number of γ/(γ′/γ′′) phase interfaces to impede dislocation movement.^[^ ^40^ ^]^

It is noteworthy that there remains room for improvement in achieving a better fit for a few data points. Those data primarily stemmed from differences in synthesis procedures and processing methods. The manufacturing information is often entirely independent of each other, rendering them unsuitable for accurate representation using conventional encoding methods. Furthermore, these methods can significantly inflate the input feature space, potentially leading to inaccuracies in ML models, particularly when dealing with high‐dimensional data and relatively small datasets.^[^ ^41^ ^]^ Nevertheless, the exploration of natural language processing techniques for addressing this challenge holds promise as a potential solution.^[^ ^42^ ^]^

## 4 Conclusion

In this study, we developed an interpretable ML approach to investigate the influence of HT processes on the creep properties of superalloys and enhanced the accuracy of creep rupture life prediction. First, we curated a representative dataset, capturing the creep performance of the alloy, which served as the foundation for predicting creep rupture life. Subsequently, a classification algorithm was employed to evaluate the impact of HT processes on creep behavior. Furthermore, the regression model provided accurate predictions of creep rupture life. Notably, the models were validated with a newly designed HT, demonstrating significant improvement compared with the previous study. In summary, our developed ML method offers an efficient and effective means to evaluate the influence of HT processes on the creep properties of superalloys while significantly improving creep rupture life prediction accuracy. The approach considers crucial factors such as HT process parameters and selected microstructural parameters to streamline the alloy design process. The enhanced creep rupture life prediction through HT leads to considerable time and cost savings.

## 5 Experimental Section

### Modeling

In the classification model, Key physical features based on process–structure–property relationships were combined with conventional classification models, evaluation of HT processes, and enhancement of model interpretability. The feature‐based screen strategy proposed in this paper aimed to obtain the key features affecting the creep rupture life. The process of obtaining the essential factors included three steps: Correlation screening, counting, and exhaustive elimination.

### Evaluation of ML Models

To verify the generalization capability of ML models, tenfold cross‐validation was performed on alloy samples. To evaluate the performance of the models, four widely used evaluation metrics: R^2^, RMSE, MAPE, and Error, were utilized as expressed in Equations ((4), (5), (6), (7)), respectively.

(4) $$R^{2}=1-\frac{\sum_{i=1}^{n}\left(y_{measured}-y_{predicted}\right)}{\sum_{i=1}^{n}\left(y_{measured}-y_{mean}\right)}$$

(5) $$RMSE=\sqrt{\frac{1}{n}\sum_{i=1}^{n}{\left(y_{predicted}-y_{measured}\right)}^{2}}$$

(6) $$MAPE=\frac{100\%}{n}\sum_{i=1}^{n}\left|\frac{y_{predicted}-y_{measured}}{y_{\mathrm{measured}}}\right|$$

(7) $$Errors=\frac{\sum_{i=1}^{n}\left(\left(y_{measured}-y_{predicted}\right)/y_{measured}\right)}{n}\times 100\%$$

where *y*_measured_ denotes the actual output, *y*_predicted_ denotes the predicted output, *y*_mean_ represents the average of the actual output, and *n* is the number of data points. R^2^ is one of the most important metrics for evaluating the accuracy of a regression model. MAPE and RMSE are other important metrics for evaluating the prediction error of models.

For classification models, the generalization ability of the models was evaluated by using tenfold cross‐validation and hold‐out methods, and the models were evaluated quantitatively using accuracy, precision, recall, Auc values, and F1‐score. Equations ((8), (9), (10), (11)) represent the definition.

(8) $$A\mathrm{ccuracy}=\frac{TP+TN}{TP+TN+FP+FN}$$

(9) $$\mathrm{Precision}=\frac{TP}{TP+FP}$$

(10) $$Recall=\frac{TP}{TP+FN}$$

(11) $$F1-score=\frac{2TP}{2TP+FP+FN}$$

*TP*, *FN*, *TN*, and *FP* are the main parameters that form the confusion matrix. The Auc value is the area under the Roc curve.

### Statistical Analysis

A significance test through Analysis of Variance (ANOVA) was conducted to validate whether there were significant differences in the assessed results of different HT on creep rupture life. By comparing the significance level (α value of 0.05), the creep rupture life data were analyzed for various HTs. The results, as detailed in Table S2 (Supporting Information), indicated that the observed differences were unlikely to be due to random factors but were more likely associated with actual variations in the evaluation process. This clearly indicated a significant impact of different HT on creep rupture life.

### Experimental Details

The experimental details of the ML method‐based reverse design optimization of HT are described in the section in Supporting Information.

## Conflict of Interest

The authors declare no conflict of interest.

## Supporting information

Supporting Information

## Data Availability

The data that support the findings of this study are available in the supplementary material of this article.
