# Supplementary material for: Interpretable Predicting Creep Rupture Life of Superalloys: Enhanced by Domain‐Specific Knowledge
Source: Adv Sci (Weinh). 2024 Jan 2;11(11):2307982. doi: 10.1002/advs.202307982 (PMC10953578; doi:10.1002/advs.202307982)
Supplement: Supplementary file 1 — Supporting Information [file ADVS-11-2307982-s001.pdf]

ADVANCED SCIENCE
Open Access

Supporting Information

for *Adv. Sci.*, DOI 10.1002/advs.202307982

Interpretable Predicting Creep Rupture Life of Superalloys: Enhanced by Domain-Specific Knowledge

Jiawei Yin, Ziyuan Rao, Dayong Wu*, Haopeng Lv, Haikun Ma, Teng Long, Jie Kang, Qian Wang, Yandong Wang and Ru Su**

Supporting Information

Interpretable predicting creep rupture life of superalloys: enhanced by domain-specific knowledge

Jiawei Yin[a], Ziyuan Rao[b*], Dayong Wu[a*], Haopeng Lv[a], Haikun Ma[a], Teng Long[c], Jie Kang[a], Qian Wang[a], Yandong Wang[d], Ru Su[a,c*]

[a]*School of Materials Science and Engineering, Hebei University of Science and Technology, Shijiazhuang, Hebei, 050018, China*
[b]*Max-Planck-Institut für Eisenforschung, 40237 Düsseldorf, Germany*
[c]*School of Materials Science & Engineering, Shandong University, Jingshi Road 17923, 250061 Jinan China*
[d]*State Key Laboratory for Advanced Metals and Materials, University of Science and Technology Beijing, Beijing 100083, China*

1.The details of datasets

Detailed data sheets can be downloaded at https://github.com/838615495/creep-rupture-life-data, the file named creep life.csv. We also provide a simplified version of the code integrated into a single Jupyter Notebook, which is easier to perform and understand. It also can be downloaded at https://github.com/838615495/creep-rupture-life-data, the files named predict creep rupture life. Ipynb and screen features.ipynb.

Twenty-one material features relevant to creep rupture life were selected as inputs, categorized into four feature sets: alloy composition, creep test conditions, heat treatment (HT) process parameters, and γ′/γ″ phases dissolution temperature. Table S1 illustrates significant numerical differences among the material features, while discrete distributions within the features can impact prediction accuracy and reduce the convergence speed of the model. Preprocessing the data before model training was necessary. To address this, we applied Min-Max normalization (Equation. (1)) to map all features to the range [0,1] and obtained new features X*. This approach mitigates

*Corresponding author:
E-mail: z.rao@mpie.de (Z.Y. Rao), wudayong_ysu@126.com (D.Y. Wu), sxru2008@163.com (R. Su).

the influence of numerical discrepancies on the predictive performance of the ML model.

$$X^* = \frac{X - X_{min}}{X_{max} - X_{min}} \quad (1)$$

X_{min} and X_{max} are the minimum and maximum values of features X, respectively. In addition, the creep rupture life distributes from 8 h to 2599 h, we treated it by logarithmic scaling as shown in Equation. (2), so that we could conveniently recover the predicted creep rupture life.

$$Y^* = log(Y) \quad (2)$$

where Y^* represents processing of creep rupture life data on a logarithmic scale and Y represents initiative creep rupture life data.

Table S1 Summary of the input material features

Index	Feature	Description	Min	Max
1	Ni	Mass percent of Ni	51.204	74.0931
2	Cr	Mass percent of Cr	12.98	20.45
3	Co	Mass percent of Co	0	15.3
4	Fe	Mass percent of Fe	0	20.215
5	Al	Mass percent of Al	0.37	3.48
6	Ti	Mass percent of Ti	0.9	5.18
7	Nb	Mass percent of Nb	0	5.48
8	Mo	Mass percent of Mo	0	9.87
9	W	Mass percent of W	0	6
10	B	Mass percent of B	0	0.03
11	C	Mass percent of C	0.015	0.08
12	Zr	Mass percent of Zr	0	0.07
13	γ'/γ''pdT	γ'/γ'' phases dissolution temperature (°C)	900	1162
14	StT	Solution treatment temperature (°C)	0	1260
15	Stt	Solution treatment time (h)	0	16
16	StaT	Stable aging temperature (°C)	0	930

Index	Feature	Description	Min	Max
17	Stat	Stable aging time (h)	0	48
18	AT	Aging temperature (℃)	0	800
19	At	Aging time (h)	0	300
20	T	Test temperature (℃)	593	900
21	S	Test stress (MPa)	25	1172

This study utilized data from previously published research, focusing on the impact of different HT processes on creep rupture life. To ensure consistency and comparability, we chose the median as the threshold for evaluation, mitigating the influence of potential outliers. Significance level analysis indicated that the data based on creep life could be distinctly categorized statistically. Simultaneously, we conducted an evaluation from the perspective of creep mechanisms, considering the impact of processes under extreme conditions and integrating process, structure, and performance, thus possessing meaningful physical significance.

2. Details of labeling

2.1 Details of labeling HT

In order to conduct an initial evaluation of the HT process and facilitate subsequent classification using ML models, as shown in Figure. S1, an evaluation of the HT process was performed from the perspectives of statistics and creep mechanisms.

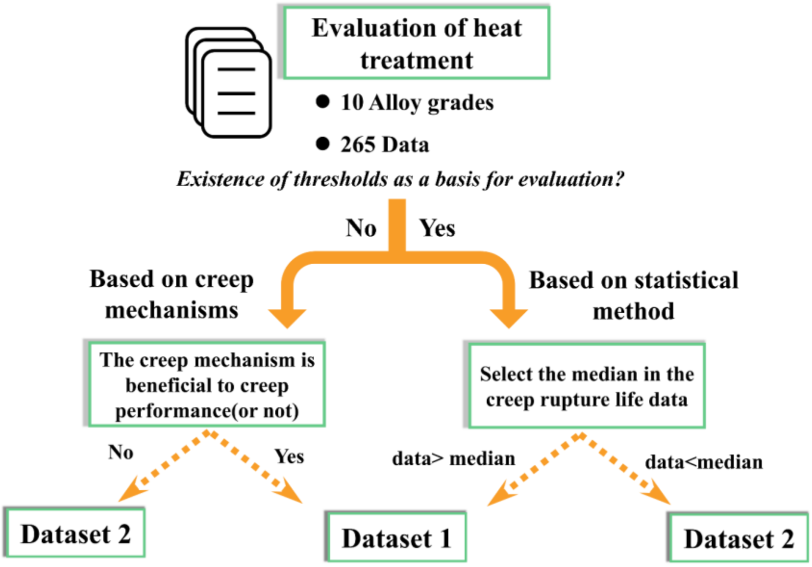

Figure. S1 The procedure of the evaluation of the HT process.

Statistics perspective: For the same superalloy grade data, the threshold value for creep rupture life is selected as the median, considering approximate test conditions. If the creep rupture life in the dataset exceeds this threshold, it indicates a positive effect of the corresponding HT process on creep rupture life under those conditions. Conversely, if the creep rupture life falls below the threshold, it suggests insignificant positive effect.

Creep mechanisms perspective: When a threshold value does not exist for some of the data as a basis for evaluation, the HT process is evaluated by analyzing whether the microstructure of the alloy is suitable for the creep test conditions. The creep test conditions for these data are mainly high temperature and low stress. When the test conditions are high temperature and low stress, the creep mechanism of the alloy is diffusional creep. Directional diffusion of the atoms under stress causes deformation of the alloy. The atoms within the alloy diffuse in two directions under tangential stress, one is diffusion along the grain boundaries to form vacancies, which in turn form micro-voids, merging along the grain boundaries to form microcracks. And the other is the diffusion of atoms within the grain, which continuously form micro-voids in the grain. Under the combined influence of these two atomic diffusion effects, the rupture mode of the alloy is intergranular rupture [1]. The creep rate (ε) of the alloy in this creep mode

can be represented by Equation. (3) (Coble creep) and Equation. (4) (Nabarro-Herring creep) : [2]

$$\varepsilon = C_1 \frac{D_V \Delta\mu}{KTd^2} \tag{3}$$

$$\varepsilon = C_2 \frac{D_b \delta \Delta\mu}{KTd^3} \tag{4}$$

where C1 and C2 are the constants. K and T are Boltzmann constant and absolute temperature. Dv and Db represent the appropriate lattice diffusion coefficient and the diffusion coefficient, respectively. μ is the chemical potential. δ is the effective width of this high-diffusivity layer. In this mode, the creep rate is directly proportional to the applied stress and inversely proportional to the grain size (d).

As shown in Figure. S2, in diffusional creep, there are primarily two deformation mechanisms:(1) At low stress and temperatures ranging from moderate to high, the predominant creep mechanism is Coble creep, where atoms diffuse along grain boundaries. (2) At low stress and higher temperatures, the primary creep mechanism shifts to Nabarro-Herring creep, where atoms migrate through vacancies, moving from regions under tensile stress to regions under compressive stress. It's noteworthy that Coble creep requires lower activation energy, making it more likely to occur, and it exhibits higher sensitivity to grain structure [2].

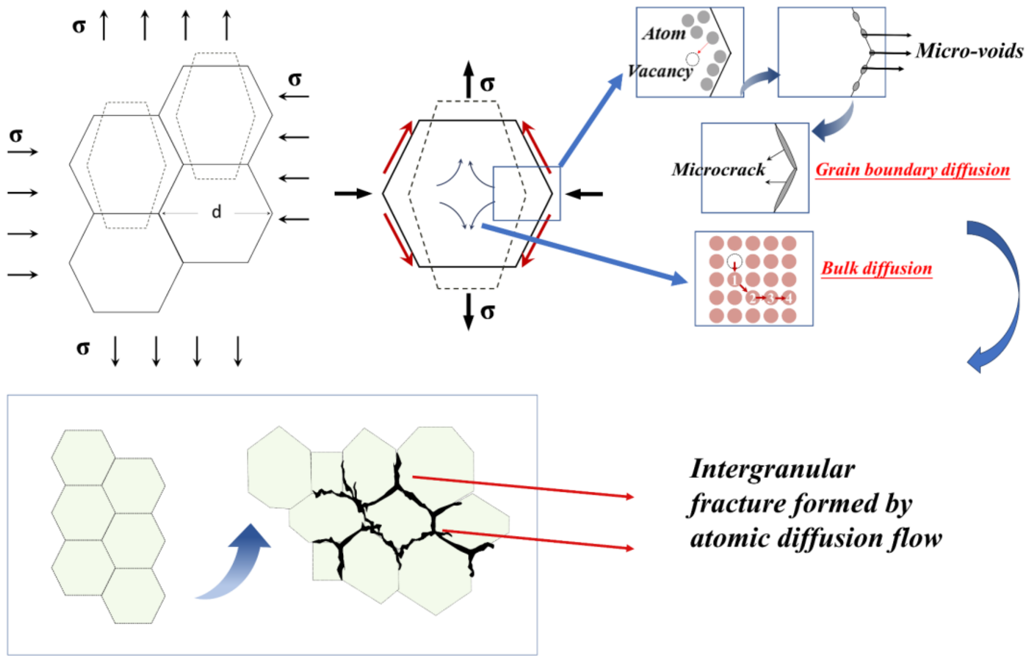

Figure S2. Grain boundary diffusion and bulk diffusion process during creep deformation with an applied, σ, in superalloys.

Therefore, within a certain size range, relatively coarse grains can improve creep performance under this mechanism. In the HT process, grain size can be controlled by adjusting the solid solution treatment temperature [3]. When the solid solution temperature exceeds the dissolution temperature of γ′/γ″ phases in the alloy and is held for an appropriate time, the γ′/γ″ phases precipitated at grain boundaries will dissolve back into the matrix, thereby losing their pinning effect on the grains. As a result, the grains will noticeably grow, eventually forming a coarser grain structure. In the collected data, there were no instances of excessively coarse grain size caused by prolonged solid solution treatment that would impair creep performance. Therefore, in cases where a threshold value is absent and diffusional creep occurs, the influence of the HT process on the creep properties can be evaluated by evaluating whether the solid solution temperature exceeds the dissolution temperature of the γ′/γ″ phases.

Through the above two methods, it is now possible to evaluate the HT processes for the majority of data. However, in the IN718 alloy dataset, there are some data that are difficult to evaluate. One of the essential roles of the HT process

of IN718 alloy is to regulate the amount of δ phase. The influence of the δ phase on

the creep performance of IN718 alloy exhibits a dual nature: its precipitation along grain boundaries can inhibit grain boundary sliding and prevent the formation and propagation of intergranular cracks [4]. However, the main strengthening phase in the alloy is γ″ phase, and excessive δ phase may hinder the formation of γ″ phase, weakening the overall strengthening effect of the alloy [5]. Additionally, the hard and brittle properties of the δ phase impede the movement of dislocations during hot deformation, promoting the initiation of grain boundary cracks. Many studies have indicated that in the high-temperature creep deformation of IN718 alloy, the induced cracking by the δ phase leads to transgranular fracture, representing the dominant creep fracture mechanism [6].

Nevertheless, within a certain range, the δ phase has a more significant positive effect on creep. Research findings suggested that when the volume fraction of δ phase precipitates is less than 3%, its positive influence on superalloys generally outweighs the negative effects [7]. Therefore, if the HT process can control the precipitation of the δ phase within 0-3%, it would be beneficial for creep performance. Utilizing the CALPHAD method, we calculated the volume fraction of δ phase for each data point in the IN718 dataset after HT and evaluated the influence of the HT on the its creep performance.

For the classified data, we conducted a significance level analysis and found that the significance level is less than 0.05, indicating that our study can significantly distinguish the data into two categories statistically. The specific significance level value is X (a specific numerical value), and the statistical testing method used is Y. This result further emphasizes the reliability of our study in classification.

Table S2 Significance analysis of classification results for each alloy dataset.

Alloy grade	Significance level is less than 0.05 or not	
Waspaloy	Yes	/
IN718	Yes	/
IN718g	Yes	/
U720Li	Yes	/
AD730	Yes	/
FGH4096	Yes	/
FGH95	Yes	/
Rene41	Yes	/
GH33A	Yes	/
GH4099	Yes	/

After we used above methods to evaluate the effect of the HT process on creep rupture life and divided the dataset into two datasets, dataset 1 and dataset 2.

2.2 Details of the results of creep rupture life prediction

We employed five regression models, including Support Vector Regression (SVR), Random Forest (RF), Gradient Boosting Regression (GBR), Multi-Layer Perceptron Regression (MLPR), and Linear Regression (LR), to predict the creep fracture life. Table 1 presents the values of RMSE and R^2 for these five different prediction models (10-fold cross-validation). From the Figure. S3 and Table S2, it is evident that the predictive outcomes of all models exhibit similar distribution trends. Simultaneously, upon analyzing the data, we found that despite the fact that the predictive outcomes of the MLPR model align more closely with our expectations, its predictive accuracy falls short. As a result, we ultimately opted for the SVR model as our preferred final predictive model.

Table S3 The comparison of prediction performance of five models on the overall dataset.

Candidate models	R^2	RMSE	Dataset 1 P>A: P<A	Dataset 2 P>A: P<A
SVR	0.676	0.535	43:97	86:39
RF	0.582	0.747	41:99	91:34
GBR	0.489	0.791	53:87	85:40
MLPR	0.488	0.787	59:81	75:50
LR	0.438	0.786	31:109	87:38

*P= Predicted values, A = Actual values.

2.3 The first step of screening method

We chose the criterion of the absolute value of the correlation coefficient (r)being greater than the median of all absolute correlation coefficients as the initial feature selection standard. This is because we believe that features with higher correlation with the target variable are more likely to positively impact the model's performance. As illustrated in the Figure S3, we determined a retention threshold (10 times) based on stability, by counting the number of times each feature was retained during model building, resulting in an initial set of 16 features. This helps ensure that our selection is robust and not influenced by randomness.

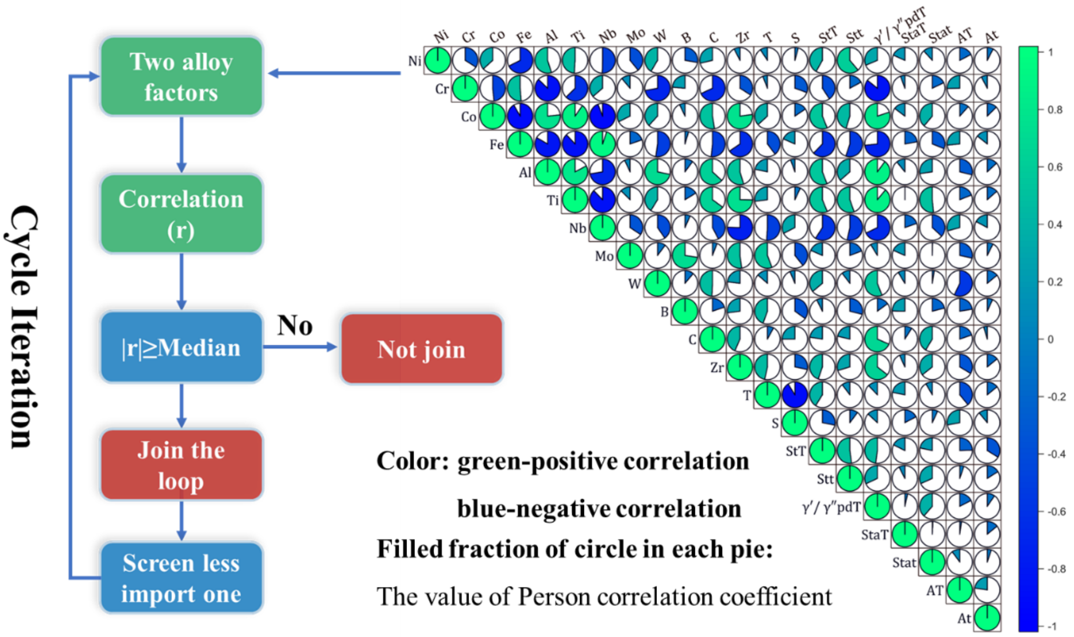

Figure S3. Feature screening workflow of correlation screening. (First step)

2.2 Transfer learning evaluate HT after labeling

Considering the inefficiencies associated with direct evaluation using supervised ML, we carefully considered the solution of Transfer Learning (TL), which we believe is effective in retaining useful features from pre-trained models for other individual tasks. We therefore adopt a new migration learning-based approach to classify HT.

Through this framework shown in Figure S4, we first pre-trained a classification model using all data from Cluster 1 to Cluster 4. After that, in the TL, we freeze the first layer of the model, and only re-train the second layer, aiming to retain useful features from the pre-trained model for better adaptation to different clusters. We commence the model training process by pre-training on an extensive dataset, encompassing Clusters 1, 2, 3, and 4. This initial phase is crucial to glean a comprehensive understanding of diverse patterns and features present in the data. Subsequently, armed with the insights gained from the pre-training phase, we embark on the fine-tuning task on Clusters 1 to 4, respectively. Table S4 shows the Hyperparameters used in the models after Bayesian optimization of 100 trials.

Figure S4. Schematic diagram of TL for evaluating HT of alloys. We use all data from Cluster 1 to Cluster 4 to train a pre-trained model. After that, in the TL, we freeze the first layer of the model, and only re-train the second layer, aiming to retain useful features from the pre-trained model for better adaptation to different clusters.

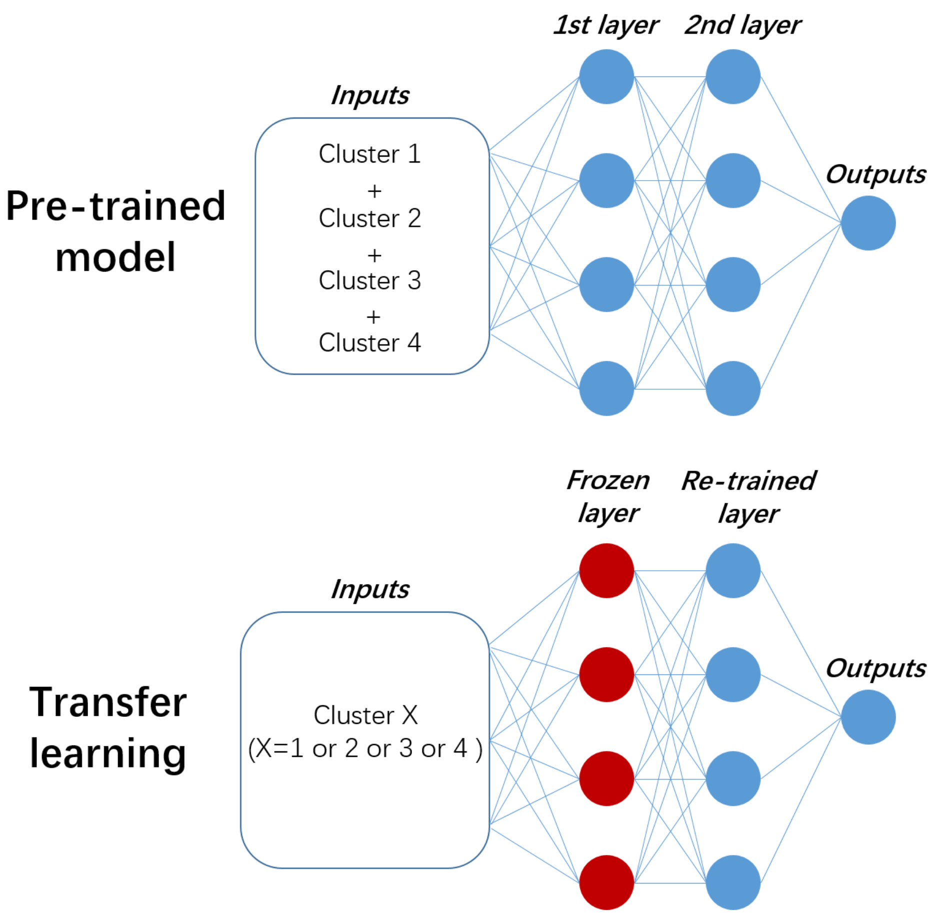

Figure S4. Schematic diagram of TL for evaluating HT of alloys. We use all data from Cluster 1 to Cluster 4 to train a pre-trained model. After that, in the TL, we freeze the first layer of the model, and only re-train the second layer, aiming to retain useful features from the pre-trained model for better adaptation to different clusters.

Table S4. Hyperparameters used in the models after Bayesian optimization of 100 trials.

Models	Learning rate	Dropout rate	Neurons in the 1st layer	Neurons in the 2nd layer	Optimizer	Activation function
Pre-trained model	0.000828	0.105079	56	56	Adam	Relu
TL models	0.0001	0.105079	56	56	Adam	Relu

Figure S5. shows the training history of the pre-trained model and TL model on Cluster 1 to 4, respectively. The pre-trained model has a quite stable loss after around 200 epochs and the accuracy gradually increases. For Cluster 1, although there is a slight increase in the validation loss, the overall accuracy is quite high and stable. For

Cluster 2, both the loss and accuracy do not change much during the training. The train and validation accuracies are both quite high. For Cluster 3, the validation loss increases and the validation accuracy is much lower than the pre-trained model. For Cluster 4, although the validation loss is quite stable, the overall validation accuracy is also much lower than the pre-trained model, with a similar behavior to Cluster 3. The summary is shown in manuscript. Overall, the behavior of TL in Cluster 1 and Cluster 2 is better than the behavior of TL in Cluster 3 and Cluster 4. We attributed this to three reasons: 1) As shown in Figure S6, the significant difference in the data size of the Clusters. The data sizes for the different Clusters are shown in Figure S5. From the data we can see that Cluster 2 has a much larger data size than the other 3 Clusters while Clusters 3 and 4 have the smallest data size. This huge bias enforces the pre-trained model learned the features mostly from Clusters 1 and 2. 2) the significant difference of the inputs of the Clusters, specifically, our model aims to evaluate the effect of HT on creep performance, involving complex physical properties such as crystal structure, lattice defects, grain boundaries, and particle boundaries. There are substantial differences among different high-temperature alloys, exemplified by IN718 (Cluster 2) alloy and Waspaloy (Cluster 1) alloy. The strengthening precipitates in IN718 alloy are pancake-shaped γ'' phases, accompanied by the significant non-coherent needle-like δ phases. In contrast, Waspaloy alloy primarily features uniformly shaped fine γ' strengthening phases. Despite similar HT processes, the significant variations in key parameters arise due to the diverse demands for microstructural control. 3) the significant difference in the intrinsic mechanism of the Clusters. When evaluating HT processes, each alloy is evaluated individually, and there is not strong correlation between different data clusters. For instance, alloys in Clusters 3 and 4 are newly developed and exhibit superior creep performance compared to alloys in Clusters 1 and 2. Therefore, with Cluster 2 dominating the dataset, even if the HT process in Cluster 3 has a negative effect on creep performance, the natural performance advantage may lead to misjudgment, causing a decrease in the TL model's performance and an increased risk of overfitting. In summary, because of the three differences of Clusters, the pre-trained

is mostly optimized to the local optimum which is based on the data of Cluster 1 and Cluster 2. When retraining the model around this local optimum, the performance of Cluster 3 and 4 naturally deteriorates.

To test our ideas, we further designed another approach based on TL to classify the HT as shown in Figure S7. In this approach, we use Cluster 2 (with the largest data points, 110), instead of all data, to train a pre-trained model. After that, in the TL, we re-train the model with Clusters 1, 3 and 4, separately. Similar to the first approach, we freeze the first layer of the model, and only re-train the second layer, aiming to retain useful features from the pre-trained model for better adaptation to different clusters. The results are shown in Table S5. From the results, compared to the former approach, the test accuracy of Cluster 2 is higher, while lower for other Clusters. This is easy to understand since we pre-trained the model only with Cluster 2. Among the other 3 Clusters, Cluster 1 has better performance than Clusters 3 and 4. This verifies our conjecture that Cluster 1 and Cluster 2 have a smaller difference in inputs and intrinsic mechanisms.

In general, we tried TF to perform classification tasks. We pre-train on the total data and then retrain on different Clusters. Compared with the approach training with different classification models, the performances of Clusters 1 and 2 are comparable, while the performances of databases 3 and 4 are obviously worse. This is due to the differences in the number, input and mechanism of the Clusters. Therefore, using TL can indeed increase the generalization ability and universality of the model. However, if there are strong differences in the different Clusters, the results will also be negatively affected. We believe that increasing the amount of data is a potential way to improve TL capabilities in the future.

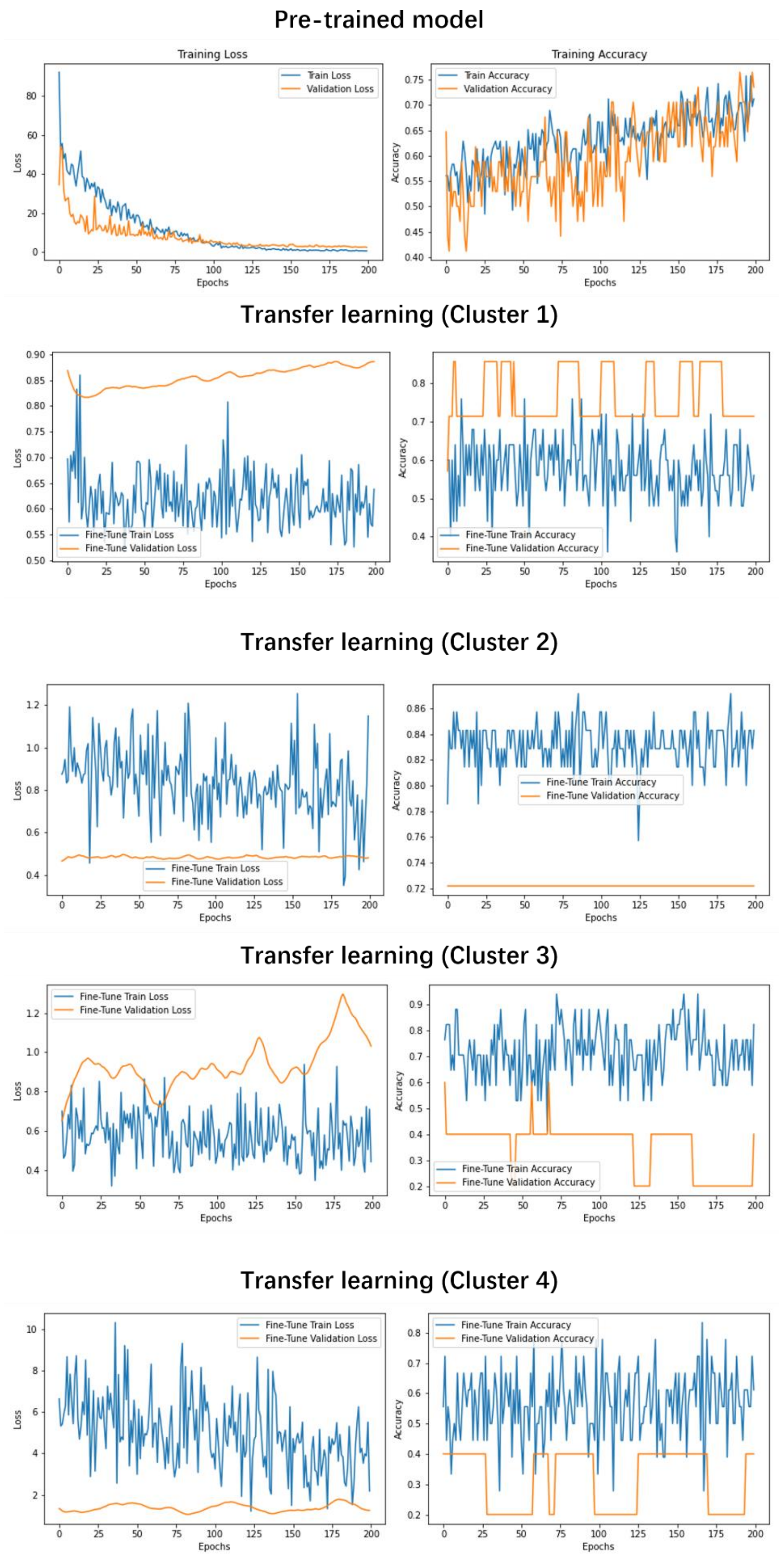

Figure S5. Training history of the pre-trained model and TL model on Cluster 1 to 4, respectively. The left and right figures show the change in the loss and accuracy with

the increase of epochs, respectively.

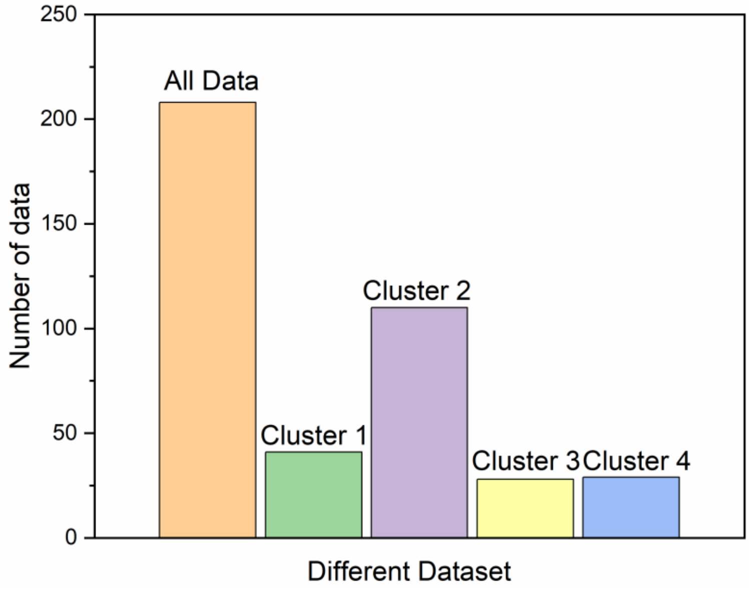

Figure S6. Number of data in each training dataset. All Data are used for pre-training and Clusters 1 to 4 are used for TL.

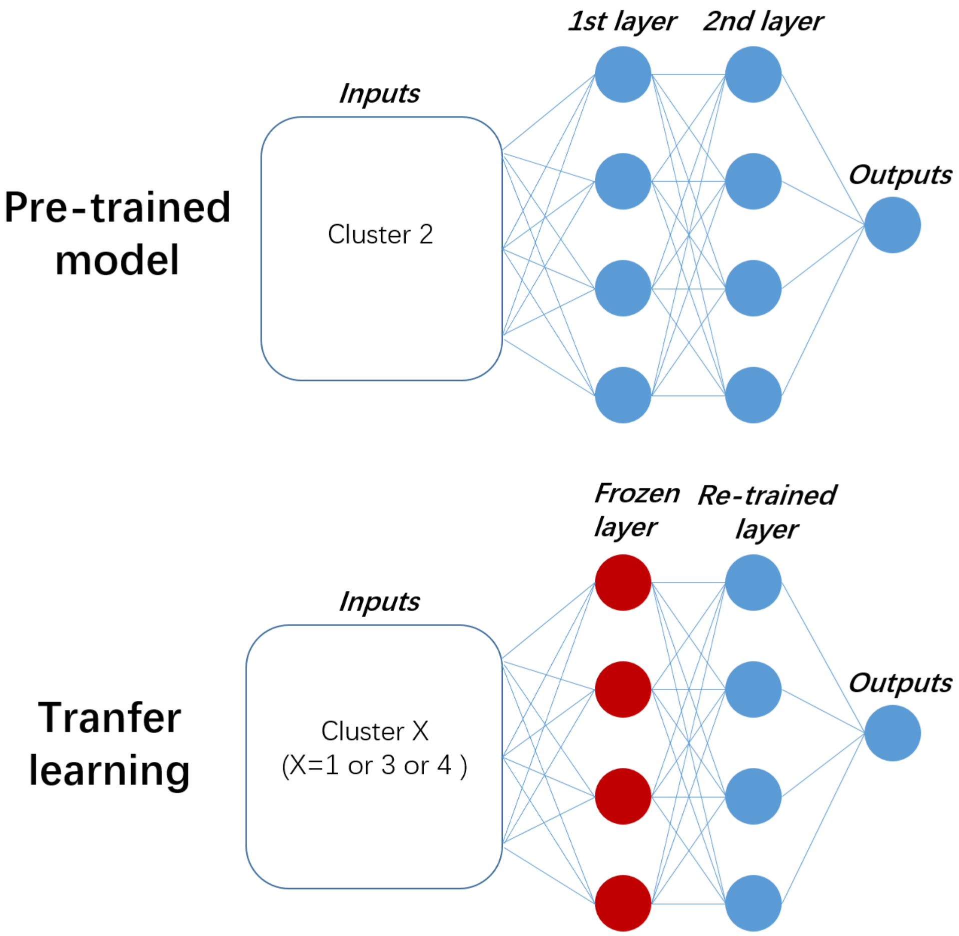

Figure S7. Schematic diagram of second TL approach for evaluating HT of alloys. In this approach, we use Cluster 2 (with the largest data points), instead of all data, to train a pre-trained model. After that, in the TL, we re-train the model with Cluster 1, 3 and 4. Similar to the first approach, we freeze the first layer of the model, and only re-train the second layer, aiming to retain useful features from the pre-trained model for better adaptation to different clusters.

Table S5. Prediction results for each cluster using Cluster 2 for the pre-training.

**Data cluster**	**models**	**Evaluation scores for the model**		
		Validation accuracy (10-fold)	Accuracy (testing set)	Auc values (testing set)
**Cluster 2**	***Pre-trained***	72.22%	81.81%	86.11%
**Cluster 1**	TL	68.90%	66.60%	61.11%
**Cluster 3**	TL	60.00%	50.00%	55.50%
**Cluster 4**	TL	50.00%	20.30%	88.90%

3. The details of evaluation of HT processes by supervised ML models

The development trajectory of nickel-based wrought superalloys is illustrated in the Figure. S8. The alloys selected in this study largely represent different stages of their development. It's worth noting that the present study does not encompass the new generation of hard-to-deformed superalloys. Currently, these new alloys still face a series of challenges, such as difficulties in microstructure control and the propensity for hot deformation-induced cracking [8]. As a result, they have not yet achieved widespread application.

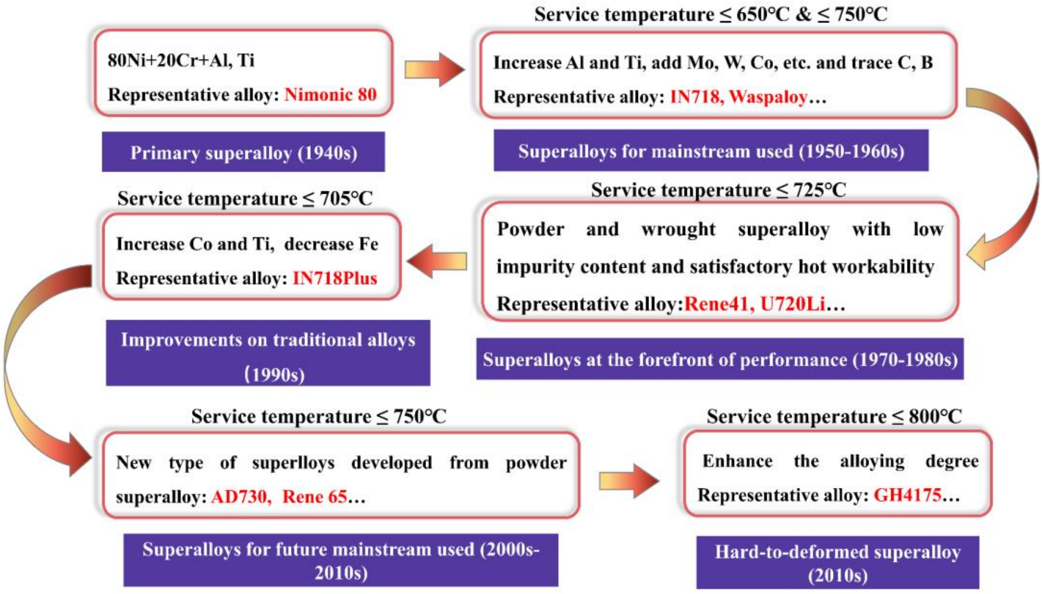

Figure S8. The development trajectory of Ni-based wrought superalloys.

The practical design of HT processes is a considerable challenge. This challenge arises due to the diverse phase transformations and microstructural variations observed in different alloy compositions during the HT process [9]. To construct a comprehensive ML model capable of predicting the effects of HT processes on creep rupture life, 208 data items were carefully selected from the total dataset. Figure. S9 illustrates the utilization of the K-Means clustering algorithm to partition the 208 data points into four clusters based on their distribution features. The data clusters from 1 to 4 represent Wasoaloy, IN718, U720Li and AD730 alloys. For each data cluster, an appropriate classification model was employed to achieve the best prediction performance. The K-Means clustering algorithm, being one of the most widely employed unsupervised learning algorithms in ML, facilitated automatic classification of the dataset into distinct categories without the need for prior training data [10].

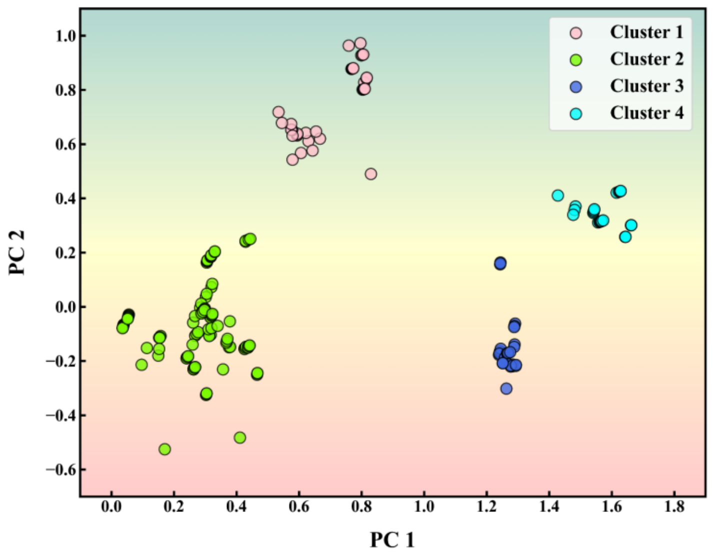

Figure S9. Two-dimensional scattering distribution of clustering results. The X-coordinate and Y-coordinate represent principal components PC1 and PC2, respectively.

The physical features added for each data cluster are presented in Table S6. There are differences in the features added to each data cluster, mainly due to the following reasons: (1) Some features were not provided in the literature due to limitations in the data sources and were difficult to supplement by simulation (e.g., grain size, cooling rate). (2) Certain features significantly assist machine learning (ML) in evaluating HT processes, reducing the need for excessive features. It should be noted that in cluster 4 (AD730 alloy), the data of the size of the γ′ phase includes secondary γ′ and tertiary γ′ phases size data.

Table S6 Features added by each data cluster

**Cluster name**	**Supersaturated solid solution (or not)**	**Cooling rate/ medium**	**Grain size**	**γ' phase size**	**V_f of δ phase**
Cluster 1	Yes	Cooling medium	Yes	Yes	No
Cluster 2	Yes	No	No	No	Yes
Cluster 3	Yes	Cooling rate	Yes	Yes	No

Cluster name	Supersaturated solid solution (or not)	Cooling rate/ medium	Grain size	γ' phase size	V_f of δ phase
Cluster 4	Yes	Cooling rate	Yes	Yes	No

***'Yes'** means that the data cluster has this feature, **'No'** means that the data cluster does not have this feature.

The confusion matrix is a tool used to measure the performance of a model in classification problems. It is a two-dimensional table used to display the relationship between the predicted results of a classification model for samples and their actual true labels. There are two class HT processes to be predicted, and the confusion matrix is used to represent the relationship between the true class and the predicted class. In the confusion matrix, the dataset 1 is positive class and dataset 2 is negative class. The correct prediction of the model is recorded as true, and the wrong prediction is recorded as false. Therefore, four basic terms can be constructed in the confusion matrix as follows. The TF, FP, FN and TN represent the number of True Positive, False Positive, False Negative and True Negative, respectively. The larger the proportion of TP and TN in the confusion matrix, the better the model prediction.

Table S7 Confusion matrix

Groundtruth	Prediction	
	Dataset 1	Dataset 2
Dataset 1	TP	FN
Dataset 2	FP	TN

True Positive (TF): the prediction by the model is dataset 1, and it is dataset 1 in groundtruth.

False Positive (FP): the prediction by the model is dataset 1, and it is dataset 2 in groundtruth.

False Negative (FN): the prediction by the model is dataset 2, and it is dataset 1 in groundtruth.

True Negative (TN): the prediction by the model is dataset 2, and it is dataset 2 in groundtruth.

As shown in tables S8 to S15 when evaluating classification models, our primary focus is on three evaluation metrics: accuracy, F1-score, and Auc value. To maintain balance between training and testing sets, our initial concern is to avoid overfitting, namely, the accuracy of the test set should be as close to or exceed the accuracy of the training set as possible. Subsequently, we pay special attention to the F1-score. Since the partitioning of the testing set may involve the use of the shuffle function, certain test sets might exhibit class imbalance issues. The utilization of the F1-score can effectively address such problems, as it comprehensively considers the predictive performance for both classes. Finally, if differentiating between models becomes challenging using the aforementioned approaches, we can resort to assessing the models based on the magnitude of the Auc value. (The ratio of the training set to the testing set is 4:1.)

Table S8 Accuracy, F1-score and Auc values of five different prediction models for cluster 1 (before adding physical features).

Prediction models	Accuracy training set	Accuracy testing set	F1-score testing set	Auc value testing set
SVC	60.95%	37.50%	0.55	0.50
**DTC**	**63.33%**	**75.00%**	**0.67**	**0.73**
RFC	63.81%	62.50%	0.67	0.70
MLPC	72.83%	50.00%	0.50	0.53
BC	78.33%	62.50%	0.67	0.70

Table S9 Accuracy, F1-score and Auc values of five different prediction models for cluster 1 (after adding physical features).

Prediction models	Accuracy training set	Accuracy testing set	F1-score testing set	Auc value testing set
SVC	63.81%	37.50	0.55	0.50
DTC	81.67%	87.50	0.80	0.83
RFC	78.33%	37.5%	0.44	0.43

**MLPC**	**82.50%**	**87.50%**	**0.86**	**0.90**
BC	78.33%	87.50%	0.86	0.90

Table S10 Accuracy, F1-score and Auc values of five different prediction models for cluster 2 (before adding physical features).

Prediction models	Accuracy training set	Accuracy testing set	F1-score testing set	Auc value testing set
SVC	89.85%	77.27%	0.80	0.77
DTC	91.52%	77.27%	0.80	0.77
RFC	79.00%	81.82%	0.86	0.80
**MLPC**	**86.36%**	**86.36%**	**0.89**	**0.87**
BC	70.50%	63.63%	0.67	0.66

Table S11 Accuracy, F1-score and Auc values of five different prediction models for cluster 2 (after adding physical features).

Prediction models	Accuracy training set	Accuracy testing set	F1-score testing set	Auc value testing set
SVC	83.01%	77.27%	0.80	0.83
**DTC**	**87.51%**	**95.45%**	**0.97**	**0.93**
RFC	80.91%	81.82%	0.86	0.83
MLPC	82.50%	86.36%	0.89	0.90
BC	85.45%	77.27%	0.71	0.76

Table S12 Accuracy, F1-score and Auc values of five different prediction models for cluster 3 (before adding physical features).

Prediction models	Accuracy training set	Accuracy testing set	F1-score testing set	Auc value testing set
SVC	69.71%	66.67%	0.67	0.67
DTC	79.22%	83.33%	0.80	0.83
RFC	75.25	66.67%	0.67	0.67
MLPC	76.71%	66.67%	0.67	0.67
**BC**	**86.00%**	**83.33%**	**0.80**	**0.83**

Table S13 Accuracy, F1-score and Auc values of five different prediction models for cluster 3 (after adding physical features).

Prediction models	Accuracy training set	Accuracy testing set	F1-score testing set	Auc value testing set
SVC	73.33%	66.67%	0.67	0.75
DTC	81.67%	83.33%	0.80	0.88
RFC	83.33%	83.33%	0.80	0.88
**MLPC**	**83.33%**	**100%**	**1.00**	**1.00**
BC	86.70%	83.33%	0.67	0.75

Table S14 Accuracy, F1-score and Auc values of five different prediction models for cluster 4 (before adding physical features).

Prediction models	Accuracy training set	Accuracy testing set	F1-score testing set	Auc value testing set
SVC	70.31%	33.33%	0.33	0.37
DTC	77.21%	50.00%	0.40	0.62
RFC	75.25	50.00%	0.57	0.50
MLPC	78.71%	50.00%	0.40	0.62
**BC**	**70.83%**	**66.67%**	**0.67**	**0.75**

Table S15 Accuracy, F1-score and Auc values of five different prediction models for cluster 4 (after adding physical features).

Prediction models	Accuracy training set	Accuracy testing set	F1-score testing set	Auc value testing set
SVC	65.99%	66.67%	0.75	0.67
DTC	78.00%	83.33%	0.80	0.83
**RFC**	**80.00%**	**83.33%**	**0.86**	**0.88**
MLPC	81.67%	50.00%	0.4	0.50
BC	86.70%	83.33%	0.80	0.83

*The bolded font in the table indicates the best model for the dataset

4. The details of improving creep rupture life prediction via screening key features

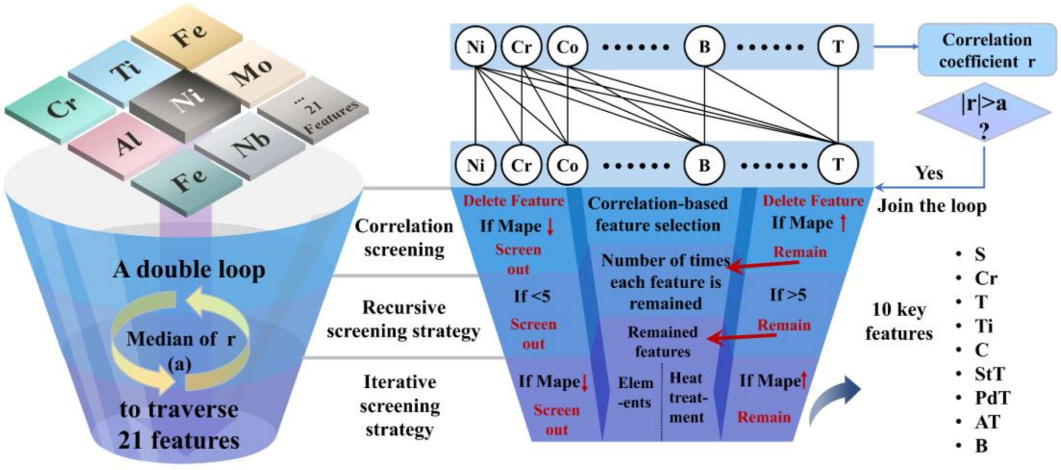

Figure S10. Steps for screening key features based on the SVR model.

As shown in figure S10, the screen steps are following:

(1) Correlation screening: The correlation coefficients between individual features were obtained, as depicted in Figure. S10, the initial step involved traversing these coefficients to identify highly correlated features. In this process, nested for-loop statements for feature traversal was utilized. We established a threshold based on the median (denoted as 'a') and evaluated the correlation coefficient (denoted as 'r') values against this threshold. Only the features with correlation coefficients surpassing the threshold ($|r| > a$) were incorporated into the regression model to assess their impact on the MAPE. Subsequently, we retained only the features that demonstrated a significant effect in reducing MAPE.

(2) Recursive screening strategy: After the completion of the nested for-loop, the retention frequency of each feature was calculated. In subsequent analyses, these features were divided into two distinct subsets to discern the effects of alloy composition and HT process parameters. Furthermore, during the calculation process, features with fewer than five occurrences were eliminated from each subset. The remaining features were then utilized for further analysis.

(3) Iterative screening strategy: In the third step of feature filtering, the experimental temperature and stress variables were retained, as they were deemed essential. The remaining 16 features underwent an iterative screening strategy based on the SVR

model. The model construction commenced with the two feature sets retained in the second step. In each iteration, one feature was gradually excluded from the input to build a new model, and this process continued until the loop condition was met. After each iteration, the features that resulted in reduced MAPE values were retained by comparing the MAPE values from the previous and current iterations. Ultimately, the ten key features that significantly influenced creep rupture life were identified.

5. The details of full-flow validation of ML approach via optimizing HT

5.1 Details of the inverse design

Our reverse design is based on the results of key feature selection, combined with an embedded physical feature classification model. Firstly, we chose Waspaloy as the raw material, considering its widespread use in current high-temperature alloy applications. Through the reverse optimization of the heat treatment process for Waspaloy, we not only further optimize performance based on its relatively reliable dataset but also facilitate the application of design experience to future newly developed alloys, especially those with compositions and service environments closely resembling it.

5.2 Experimental details

The material investigated in this study is a Waspaloy forged rod provided by AVIC Shaanxi Aero Electric Co., Ltd. The chemical composition of Waspaloy is same with collected data. Following HT, the roughly machined specimens were further processed to create fine creep test specimens, with their geometric details illustrated in Figure S11. Creep tests were conducted on a creep testing machine (CX-DS-04) equipped with an extensometer system to monitor specimen strain. Creep elongation was measured using dual extensometers and extension rods with thermocouples, installed on the shoulders of the specimen and extending to fixtures outside the furnace. All test specimens were subjected to conditions of 650°C and 780 MPa. Strain measurements during creep tests were recorded at 5-minute intervals until specimen fracture. To determine the creep

limit concerning time and strain at a specific temperature, the relationship curve between time and strain was computed using the creep test data exported by the software.

For the preparation of microstructure characterization, longitudinal cross-sections near the fracture appearance of all specimens were prepared using electrical discharge machining. After a series of grinding and polishing steps, fracture and microstructure were observed using scanning electron microscopy (SEM). Fractured surfaces of creep specimens were cleaned in acetone and alcohol through ultrasonic cleaning, and the morphology of the fractures was observed via SEM.

The creep fracture pattern of the style is shown in Figure S11. It can be clearly seen that the fracture under these test conditions is ductile fracture. The presence of more prolific cracks on the fracture surface and the wedge-shaped cracks extending along the grain boundaries and reaching the three-phase point indicate that the fracture mechanism is intergranular rupture. This is due to the fact that the grain boundaries are weakened at high temperatures, and the strength is lower than that in the matrix, and the strength of the primary γ' phase in the matrix. Transcrystalline rupture also occurred and revealed tear ridges. In summary, the fracture mode of Waspaloy in this state is a combination of intergranular and transgranular fracture.

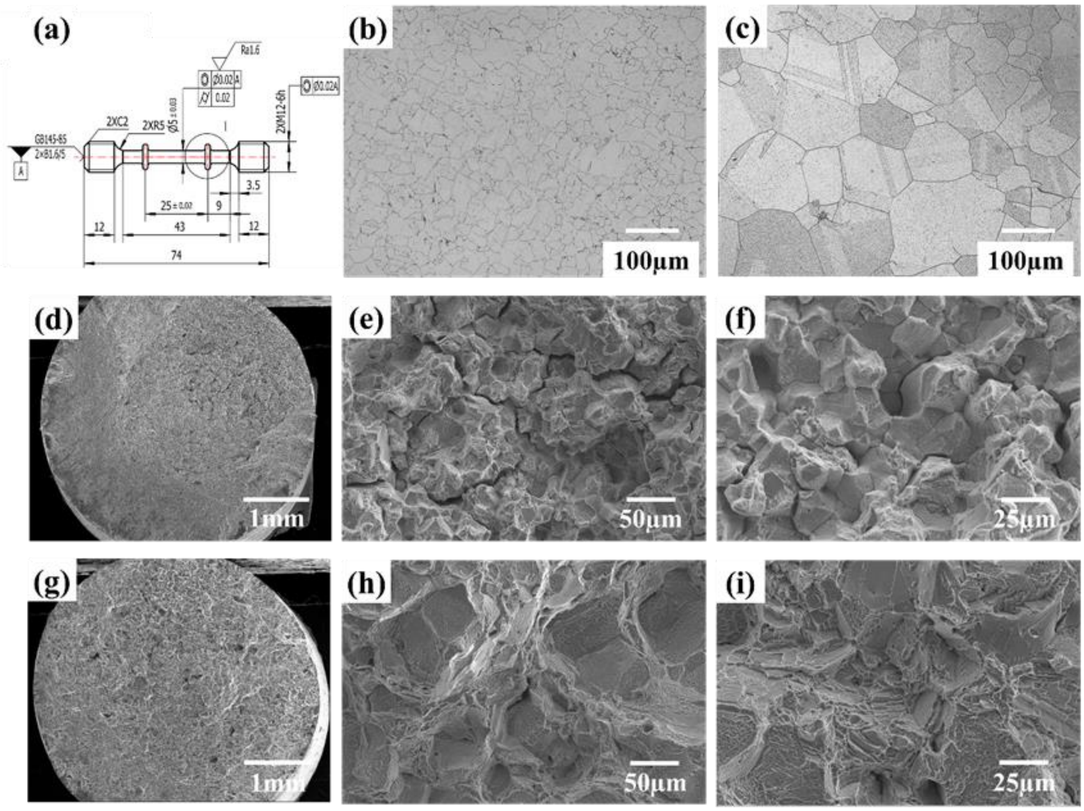

Figure S11. (a) Geometric dimension of specimens for the creep tests. (b) 1030℃ specimen (c) 1040℃ specimen. SEM images with different fracture surface magnifications, (d-f) 1030°C specimen and (g-i) 1040°C specimen.

6. The details of grain size prediction

In the acquisition of crucial physical features like grain size, reliance often lies on experimental observations or numerical simulations, such as Molecular Dynamics (MD) simulations [11], Monte Carlo simulations (MC) [12], or phase field simulations [13]. Yet, traditional experimental methods suffer from time-consuming procedures and high costs, while numerical simulation methods encounter limitations including computational complexity or resource demand. In contrast, ML has made significant strides in predicting relevant feature, owing to its advantages in terms of time efficiency and predictive performance. Integrating ML into the prediction of grain size holds promise for enhancing the predictive capabilities of material properties.

The research data for this section are derived from 11 published research articles, encompassing a total of 119 sets of data concerning the variation in grain size of

superalloys after undergoing HT. Each dataset includes information about the alloy composition, HT process parameters, initial grain size, and target feature.

The predictive results using the SVR model are shown in the Figure. S12. The ML model exhibits remarkable predictive performance, with outliers mainly concentrated in data points where grain sizes are larger than 300μm. In the classification of grain size levels, grains larger than 300μm are categorized as M-0 level; however, such excessively coarse grains are scarcely encountered in practical applications [14], thus having minimal impact on the model. Through model construction, we are able to achieve accurate predictions of grain sizes, utilizing them as inputs for forecasting HT processes as evaluated in this study using the ML approach. With the improvement of this approach in the future, the grain sizes of any superalloy after HT can be obtained and inputted as features.

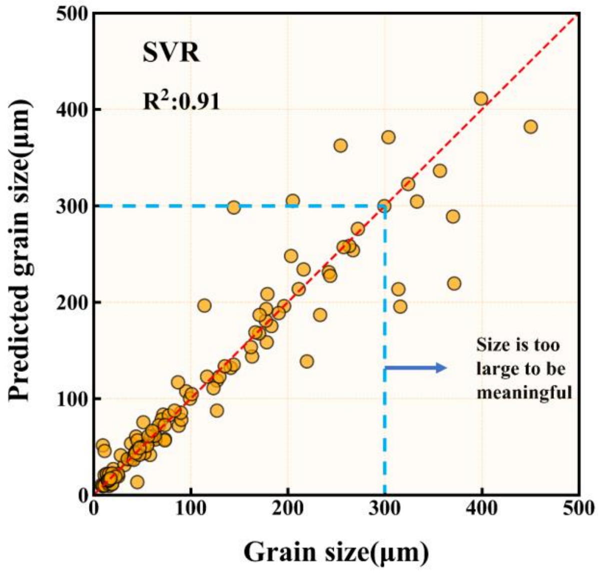

Figure S12. Predictive performance of the SVR model on grain size (10-fold cross-validation).

In order to visually analyze the impact of key features, we employed the SHAP method to assess the influence of each key feature on the prediction outcomes, as shown in the Figure. S13. We sorted the primary key features by mean value of |SHAP values|, resulting in the following order: StT > Stt > Cr > Ti > Zr > initial grain size. This suggests that higher solid solution temperatures and longer times lead to larger grain

sizes. The remaining key features are alloy compositions, which represent internal factors affecting grain size. The roles of Cr and Ti have been extensively analyzed in the main text. As for Zr, its primary role in superalloys is to facilitate the formation of larger carbides, significantly altering the types and distribution of oxides [15], thereby weakening the pinning effect of strengthening phases on grain boundaries.

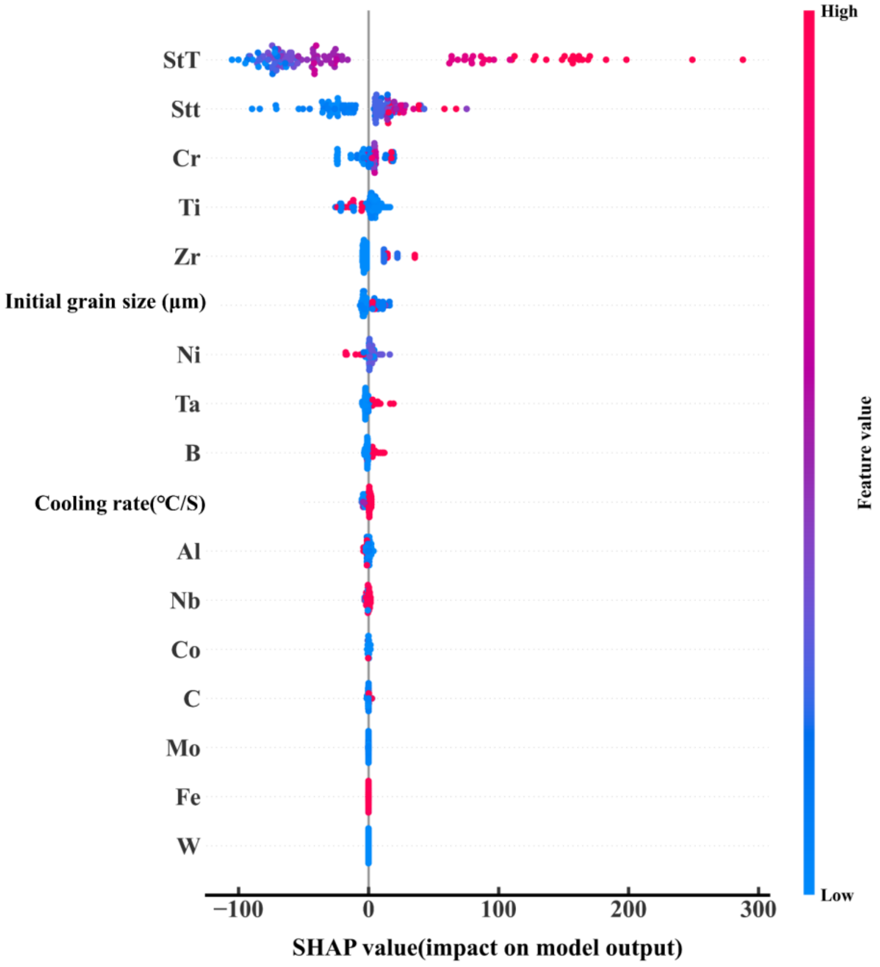

Figure S13. Interpretability analysis of the regression model for grain size (SHAP value summary plot).

In this study, we utilized two apps capable of extracting data from images, namely "get data" and "Image Pro". In cases where certain physical features data were missing (such as grain size and γ′/γ″ phases sizes), we manually captured the data using the app to ensure completeness. All the algorithms covered mentioned above are implemented in Python and scikit-learn toolkit and CALPHAD method is implemented by Thermo-Calc.

Reference

[1] M. Sattar, A. R. Othman, S. Kamaruddin, M. Akhtar, R. Khan, *Eng. Failure Anal.* **2022**, *134*, 105968.

[2] M. F. Ashby, *Surf. Sci.* **1972**, *31*, 498,.

[3] Z. Zhou, R. Zhang, C. Cui, Y. Zhou, X. Sun, *Mater. Sci. Eng. A* **2022**, *853*, 143741.

[4] W. Chen, M. C. Chaturvedi, *Acta Mater.* **1997**, *45* (7), 2735.

[5] R. Ran, Y. Wang, Y.-x. Zhang, F. Fang, H.-s. Wang, G. Yuan, G.-d. Wang, *Mater. Sci. Eng. A* **2020**, *793*, 139860.

[6] Q. Wang, S. Ge, D. Wu, H. Ma, J. Kang, M. Liu, T. Wang, B. Narayanaswamy, R. Su, *Mater. Sci. Eng. A* **2022**, *857*, 143859.

[7] J. Du, X. Lu, Q. Deng, *Rare Met. Mater. Eng.* **2014**, *43*.

[8] D. J. IANG He, ZHANG Maicang, YAO Zhihao, *Aeronaut. Manuf. Technol.* **2021**, *64* (1), 12.

[9] L. Zhang, Y. Li, Q. Zhang, S. Zhang, *Mater. Sci. Eng. A* **2022**, *844*, 142947.

[10] G. Tzortzis, A. Likas, *Pattern Recognit.* **2014**, *47* (7), 2505.

[11] V. V. Borovikov, M. I. Mendelev, T. M. Smith, J. W. Lawson, *Int. J. Plast.* **2023**, *166*, 103645.

[12] D. Leidermark, D. Aspenberg, D. Gustafsson, J. Moverare, K. Simonsson, *Comput. Mater. Sci.* **2012**, *51* (1), 273.

[13] P. E. L'vov, R. T. Sibatov, V. V. Svetukhin, *Mater. Today Commun.* **2023**, *35*, 106209.

[14] X. Wang, Z. Huang, B. Cai, N. Zhou, O. Magdysyuk, Y. Gao, S. Srivatsa, L. Tan, L. Jiang, *Acta Mater.* **2019**, *168*, 287.

[15] P. Wang, W. Song, S. Yang, J. Yang, J. Liang, J. Li, Z. Yang, Y. Zhou, X. Sun, *J. Alloys Compd.* **2023**, *965*, 171371.
